# Recessive mutations in *POLR1C* cause a leukodystrophy by impairing biogenesis of RNA polymerase III

**DOI:** 10.1038/ncomms8623

**Published:** 2015-07-07

**Authors:** Isabelle Thiffault, Nicole I. Wolf, Diane Forget, Kether Guerrero, Luan T. Tran, Karine Choquet, Mathieu Lavallée-Adam, Christian Poitras, Bernard Brais, Grace Yoon, Laszlo Sztriha, Richard I. Webster, Dagmar Timmann, Bart P. van de Warrenburg, Jürgen Seeger, Alíz Zimmermann, Adrienn Máté, Cyril Goizet, Eva Fung, Marjo S. van der Knaap, Sébastien Fribourg, Adeline Vanderver, Cas Simons, Ryan J. Taft, John R. Yates III, Benoit Coulombe, Geneviève Bernard

**Affiliations:** 1 Department of Neurology and Neurosurgery, McGill University, Department of Medical Genetics, Montreal Children's Hospital, Research Institute of the McGill University Health Center, 1001 boul Décarie, Montreal, Quebec H4A 3J1, Canada.; 2 Service de Génétique, Centre Hospitalier Universitaire Sainte-Justine, 3175 Chemin de la Côte-Sainte-Catherine, Montreal, Quebec H3T1C5, Canada.; 3 Center for Pediatric Genomic Medicine, Children's Mercy Hospital, 2420 Pershing Road, Suite 421, Kansas City, Missouri 64108, USA.; 4 Department of Child Neurology, VU University Medical Center, Neuroscience Campus Amsterdam, Amsterdam 1081 HZ, The Netherlands.; 5 Translational Proteomics Laboratory, Institut de recherches cliniques de Montréal (IRCM), 110 avenue des Pins ouest, Montréal, Québec H2W 1R7, Canada.; 6 Neurogenetics of Motion Laboratory, Montreal Neurological Institute, 3801 University Street, McGill University, Montreal, Quebec H3A 2B4, Canada.; 7 Department of Chemical Physiology, The Scripps Research Institute, 10550 North Torrey Pines Road SR302, La Jolla, California 92037, USA.; 8 Division of Neurology and Clinical and Metabolic Genetics, the Hospital for Sick Children, University of Toronto, 555 University Avenue, Toronto, Ontario M5G 1X8, Canada.; 9 Department of Paediatrics, Faculty of Medicine, University of Szeged, Temesvári krt. 35-37, Szeged H-6726, Hungary.; 10 T.Y. Nelson Department of Neurology and Neurosurgery, The Children's Hospital at Westmead, Locked Bag 4001, Westmead, New South Wales 2145, Australia.; 11 Institute for Neuroscience and Muscle Research, The Children's Hospital at Westmead, Locked Bag 4001, Westmead New South Wales 2145, Australia.; 12 Department of Neurology, University Clinic Essen, University of Duisburg-Essen, Hufelandstrasse 55, 45147 Essen, Germany.; 13 Department of Neurology, Donders Institute for Brain, Cognition, and Behaviour, Radboud University Medical Center, PO Box 9101, Nijmegen 6500 HB, The Netherlands.; 14 Department of Pediatrics and Adolescent Medicine, Deutsche KlinikfürDiagnostik, Wiesbaden 65191, Germany.; 15 Department of Neurosurgery, Faculty of Medicine, University of Szeged, 6 Semmelweis Street, Szeged H-6725, Hungary.; 16 Service de Génétique, Hôpital Pellegrin, CHU Bordeaux and University Bordeaux, Laboratoire MRGM (EA4576), Bordeaux 33076, France.; 17 Department of Paediatrics, The Chinese University of Hong Kong, Prince of Wales Hospital, Shatin, Hong Kong, SAR China.; 18 Université de Bordeaux, Institut Européen de Chimie et Biologie, ARNA Laboratory, Pessac F-33607, France.; 19 Institut National de la Santé Et de la Recherche Médicale, INSERM—U869, ARNA Laboratory, Bordeaux F-33000, France.; 20 Center for Genetic Medicine Research, Children's National, 111 Michigan Avenue Northwest, Washington, District of Columbia 20010, USA.; 21 Department of Neurology, Children's National, 111 Michigan Avenue Northwest, Washington, District of Columbia 20010, USA.; 22 George Washington University, School of Medicine, Washington, District of Columbia 20052, USA.; 23 Institute for Molecular Bioscience, University of Queensland, Brisbane, Queensland 4072, Australia.; 24 Departments of Integrative Systems Biology and Pediatrics, School of Medicine and Health Sciences, The George Washington University, Washington, District of Columbia 20037, USA.; 25 Illumina Inc., 5200 Illumina Way, San Diego, California 92122, USA.; 26 Department of Biochemistry, Université de Montréal, Pavillon Roger-Gaudry, CP 6128, Succ Centre-Ville, Montreal, Québec H3C 3J7, Canada.

## Abstract

A small proportion of 4H (Hypomyelination, Hypodontia and Hypogonadotropic Hypogonadism) or RNA polymerase III (POLR3)-related leukodystrophy cases are negative for mutations in the previously identified causative genes *POLR3A* and *POLR3B*. Here we report eight of these cases carrying recessive mutations in *POLR1C*, a gene encoding a shared POLR1 and POLR3 subunit, also mutated in some Treacher Collins syndrome (TCS) cases. Using shotgun proteomics and ChIP sequencing, we demonstrate that leukodystrophy-causative mutations, but not TCS mutations, in *POLR1C* impair assembly and nuclear import of POLR3, but not POLR1, leading to decreased binding to POLR3 target genes. This study is the first to show that distinct mutations in a gene coding for a shared subunit of two RNA polymerases lead to selective modification of the enzymes' availability leading to two different clinical conditions and to shed some light on the pathophysiological mechanism of one of the most common hypomyelinating leukodystrophies, POLR3-related leukodystrophy.

## Abstract (web summary)

Some RNA polymerase (POLR) 3-related leukodystrophy cases do not have the causal mutations in *POLR3A* and *POLR3B*. Here, by exome sequencing, the authors identify recessive mutations in *POLR1C*, a gene encoding a shared POLR1 and POLR3 subunit, impairing assembly and nuclear import of POLR3, but not POLR1.

## Graphical abstract

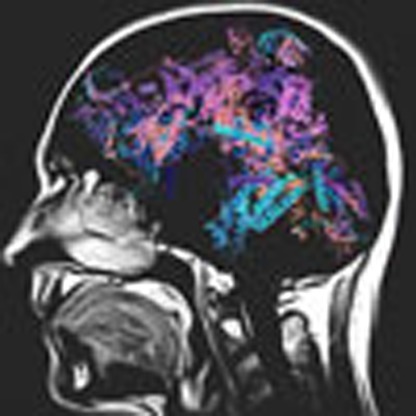

Leukodystrophies are a heterogeneous group of genetically determined disorders characterized by abnormal white matter on brain imaging^1^,^2^. They are classified as hypomyelinating and non-hypomyelinating leukodystrophies based on magnetic resonance imaging (MRI) characteristics^2^, depending on whether the principal problem appears to be a lack of myelin deposition during development or altered myelin homeostasis. RNA polymerase III (POLR3)-related leukodystrophy or 4H (Hypomyelination, Hypodontia and Hypogonadotropic Hypogonadism) leukodystrophy (MIM 607694, 614381)^3^ was found to be caused by recessive mutations in *POLR3A* (MIM 614258) or *POLR3B* (MIM 614366)^4^,^5^,^6^,^7^,^8^,^9^ and is characterized by an expanding spectrum of clinical^3^,^10^ and radiological features^10^,^11^,^12^. POLR3A and POLR3B are, respectively, the largest and second largest of the 17 subunits that constitute POLR3. Together, they form the catalytic centre of the enzyme. POLR3 synthetizes small non-coding RNAs, including tRNAs, 5S RNA, 7SK RNA and U6 RNA, that are involved in the regulation of essential cellular processes, including transcription, RNA processing and translation^13^. A subset of patients (∼5%) presenting with compatible clinical and/or radiological features of POLR3-related leukodystrophy have no detectible mutations in either *POLR3A* or *POLR3B,* suggesting that mutations in one or more additional genes may result in this presentation. We hypothesized that genes coding for other POLR3 subunits or for proteins interacting with POLR3 would be strong candidates in these cases.

In this study, we identified recessive mutations in *POLR1C*, a gene encoding for a subunit common to POLR1 and POLR3, which has thus far been known only to be associated with autosomal recessive Treacher Collins syndrome (TCS)^14^. We also demonstrated that the leukodystrophy-causing mutations affect POLR3, but not POLR1 assembly and nuclear import, leading to decrease binding to POLR3 target genes, whereas one TCS mutation leads to normal assembly of both polymerases, but rather affects POLR1 targeting to the nucleolus, the site of POLR1 gene transcription.

## Results

### Whole-exome and Sanger sequencing for gene identification

**Figure 1:**
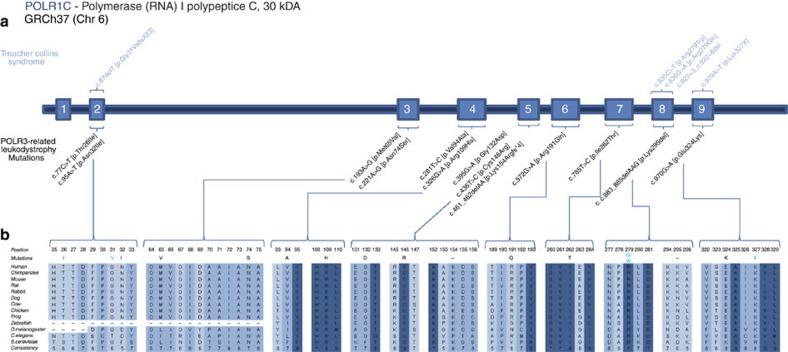
*POLR1C* mutations in leukodystrophy and TCS cases. (**a**) Genomic organization of *POLR1C* in humans (UCSC Genome Browser hg19): mutations and their positions within the *POLR1C* gDNA; in light blue are mutations that cause TCS, mutations in black cause POLR3-related leukodystrophy. (**b**) *POLR1C* mutations in patients with leukodystrophy affect amino acids that are conserved through species.

**Figure 2:**
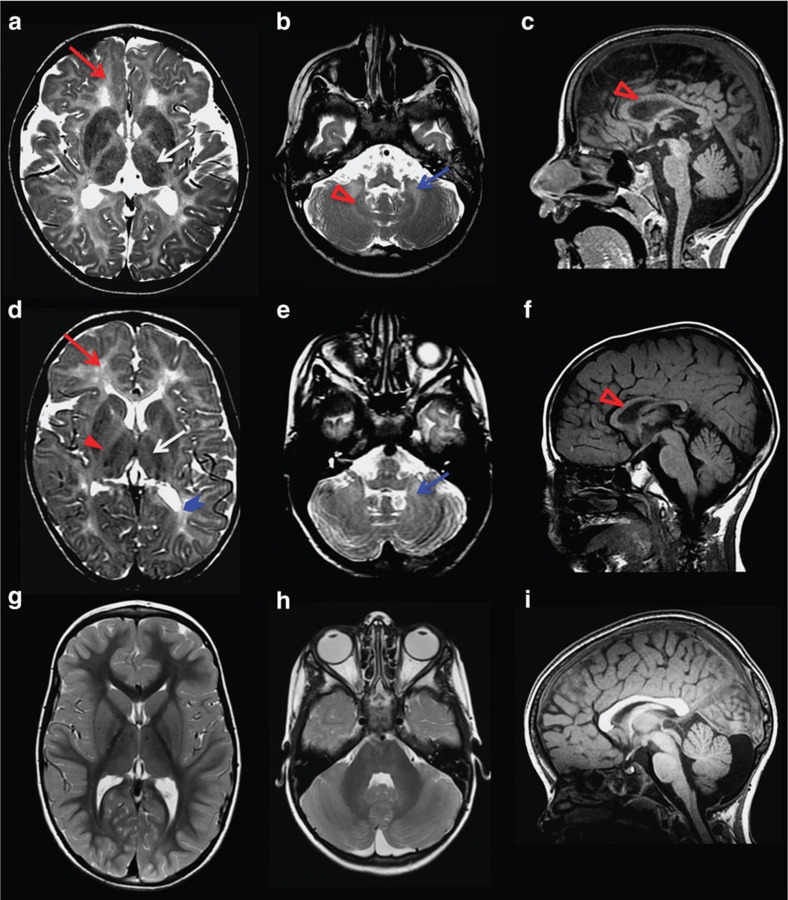
MRI characteristics of POLR3-related leukodystrophy caused by *POLR1C* mutations. Axial T2-weighted (**a**,**b**,**d**,**e**,**g**,**h**) and sagittal T1-weighted (**c**,**f**,**i**) images of case 1 aged 6 years (**a–c**) and case 2 aged 4.5 years (**d–f**) compared with a healthy control aged 4 years (**g–i**). Diffuse hyperintense signal of the supratentorial (red arrow, **a,d**) and cerebellar (blue arrow, **b,e**) white matter is visible on the T2-weighted images, indicating hypomyelination. There is no cerebellar atrophy. As typical for POLR3-related leukodystrophy, the ventrolateral thalamus (white arrow, **a,d**), the optic radiation (thick arrowhead blue, **d**) and the dentate nucleus (open red arrowhead, **b**) show a relative hypointense signal on the T2-weighted images resulting in an easily visible dentate nucleus (**b**) as compared with the control (**h**) as well as a small dot in the posterior limb of the internal capsule (red arrowhead, **d**). The corpus callosum is slightly thinned in case 1 and thinned in case 2 (open red arrowhead, **c,f**).

To investigate the genetic aetiology of these unexplained cases, we performed exome sequencing in three cases with typical clinical and/or radiological features of POLR3-related leukodystrophy negative for *POLR3A* or *POLR3B* mutation. Genome coverage for these three cases exceeded 56 × (Supplementary Table 1) and yielded more than 4.6 million variants per genome. Review of the data using in-house filters revealed a homozygous mutation in *POLR1C* (NM_203290; GRCh37/hg19) in two patients (c.221A>G (p.Asn74Ser) and c.95A>T (p.Asn32Ile), respectively), and compound heterozygous mutations in one (c.436T>C (p.Cys146Arg) and c.883_885delAAG (p.Lys295del)), all consistent with autosomal recessive inheritance (Fig. 1, Supplementary Table 1). These four variants were validated using Sanger sequencing. Co-segregation analysis confirmed that the mutations were inherited from heterozygous carrier parents. These variants were not observed in over 6,500 individuals of European and African American ancestry in the NHLBI Exome Sequencing Project database, in the ExAc data set nor in our internal variant databases. Furthermore, these variants were not present in more than 300 Centre d'Etude du Polymorphisme Humain (CEPH) control chromosomes. *In silico* analysis predicted those to be disease-causing (Supplementary Table 2c). These variants were found to affect highly conserved amino-acid residues (Fig. 1). To identify additional mutations, we sequenced all exons, exon–intron boundaries and 3′ and 5′ untranslated repeat of *POLR1C* (see Supplementary Table 3 for primers) in 16 other individuals selected on the basis of clinical (compatible neurological features with at least one non-neurological feature such as dental abnormalities or hypogonadotropic hypogonadism, together with hypomyelination on MRI) and/or radiological characteristics (typical MRI features of 4H leukodystrophy)^3^,^10^,^11^ but negative for *POLR3A* and *POLR3B* mutations and uncovered five additional compound heterozygous or homozygous cases (Supplementary Table 2, Fig. 1). Sequencing of the mutated exons was performed in family members for whom DNA was available to confirm segregation. In total, 13 *POLR1C* mutations were detected in eight cases (Supplementary Table 2a, Fig. 1). Similar to what is observed in cases with the disease caused by mutations in *POLR3A* or *POLR3B*, clinical and radiological characteristics of these eight cases were compatible with POLR3-related leukodystrophy; however, patients did not necessarily have all clinical (that is, neurological, dental, ophthalmological and endocrine abnormalities) and MRI features^1^,^3^,^12^ (Table 1, Fig. 2, Supplementary Tables 4 and 5) of the disease.

### Impact of *POLR1C* recessive mutations on POLR1 and POLR3

**Figure 3:**
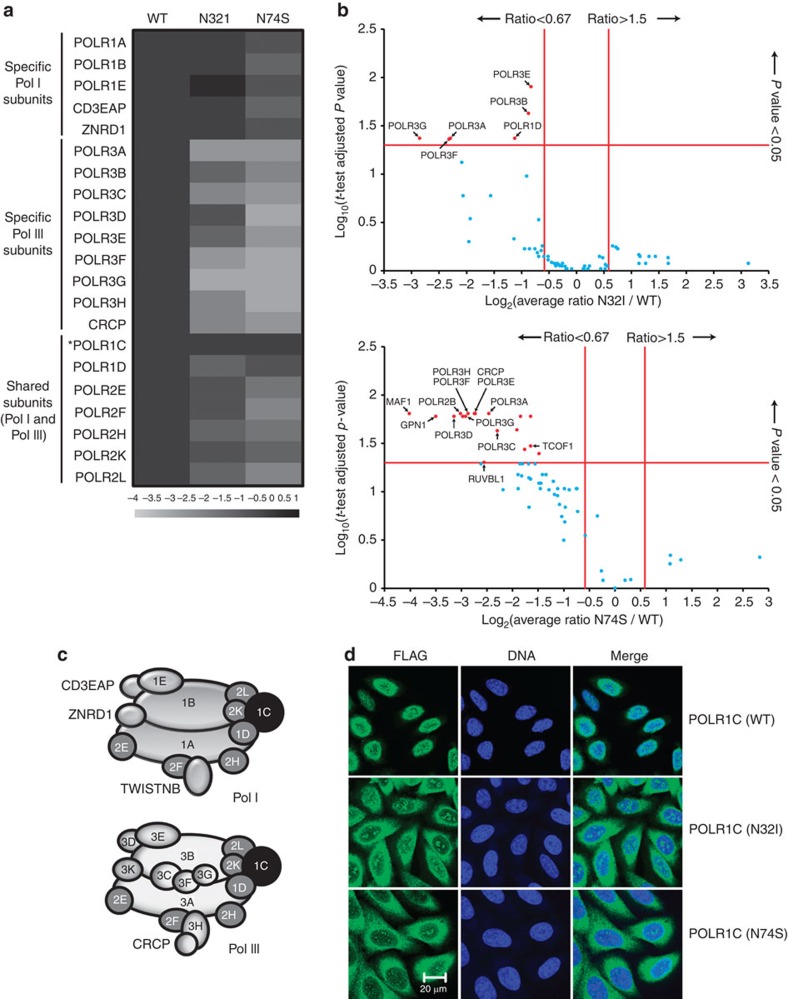
Impact of *POLR1C* mutations on polymerase assembly and nuclear import. (**a**) FLAG-tagged POLR1C variants, either the wild-type (1C) polypeptide or mutated versions having a N32I or a N74S substitution, were expressed in HeLa cells and purified using anti-FLAG affinity chromatography. The co-purified proteins were identified using LC-MS/MS mass spectrometry. The heatmap contains the log_2_-transformed average spectral count ratios N32I or N74S/WT across all three replicates. Spectral counts were computed with Mascot (see Supplementary Table 6 for the complete data set). Specific and shared POLR1 (Pol I) and POLR3 (Pol III) subunits are identified on the left. POLR1C (the bait) is identified by an asterisk. (**b**) Volcano plots of the log_2_-transformed average spectral count ratios N32I or N74S/WT (*x* axis) and the –log_10_-transformed *P* values (adjusted with the Benjamini–Hochberg procedure) resulting from the two-tailed one-sample *t*-tests of the high-confidence interactors of POLR1C. Red proteins show a level of differential interaction with POLR1C that is statistically significant, while blue proteins do not. (**c**) Schematic representation of the subunit composition of POLR1 (Pol I) and POLR3 (Pol III; see refs 53 for details). Shared subunits are in grey and POLR1C in black. (**d**) Immunofluorescence experiments showing the cellular localization of tagged POLR1C variants. Nuclei are stained using TO-PRO-3 iodine. Scale bar, 20 μm.

**Figure 4:**
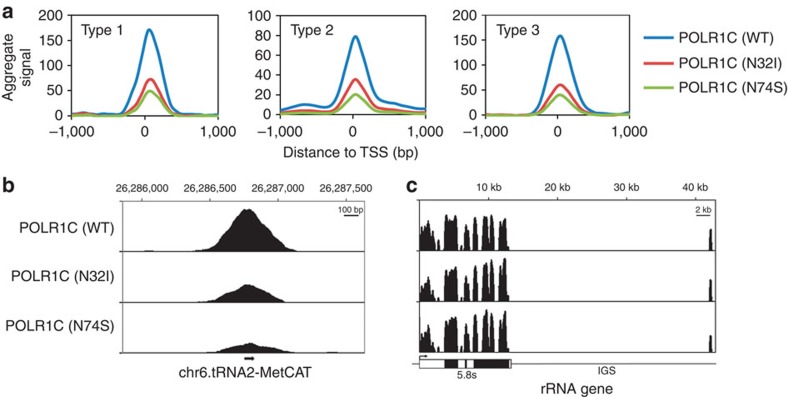
Impact of *POLR1C* mutations on polymerase association with chromatin. (**a**) ChIP-Seq experiments of FLAG-tagged POLR1C variants (wild type, N32I or N74S). Aggregate profile produced with the annotation mode of the Versatile Aggregate Profiler shows ChIP-Seq data sets over the three classes of POLR3-transcribed genes, as defined by the promoter structure. Type 1 genes have an internal promoter composed of A and C boxes (5S rRNA genes). Type 2 genes have an internal promoter composed of A and B boxes (tRNA genes, for example), while the promoter of type 3 genes (U6, 7SK, RNase P and others) is located upstream of the transcription start site (TSS)^55^. The TSS was used as the reference point. (**b**) IGV view of a tRNA-Met gene transcribed by POLR3. POLR1C binding is decreased in mutated variants compared with wild type. IGS, intergenic spacer. (**c**) IGV view of one ribosomal DNA (rDNA) repeat. The rDNA gene encodes a 45S pre-rRNA precursor that will generate the 5.8S, 18S and 28S rRNAs. There are ∼400 copies of the rDNA gene arranged in tandem repeats in the human genome. rDNA repeats are not present in the reference genome assemblies; therefore, unique reads were aligned directly to the human rDNA reference sequence (NCBI accession number: HSU13369)^56^. No differences were observed in POLR1C occupancy between wild-type and mutant variants. A schematic of a rDNA repeat is included below the graph.

**Figure 5:**
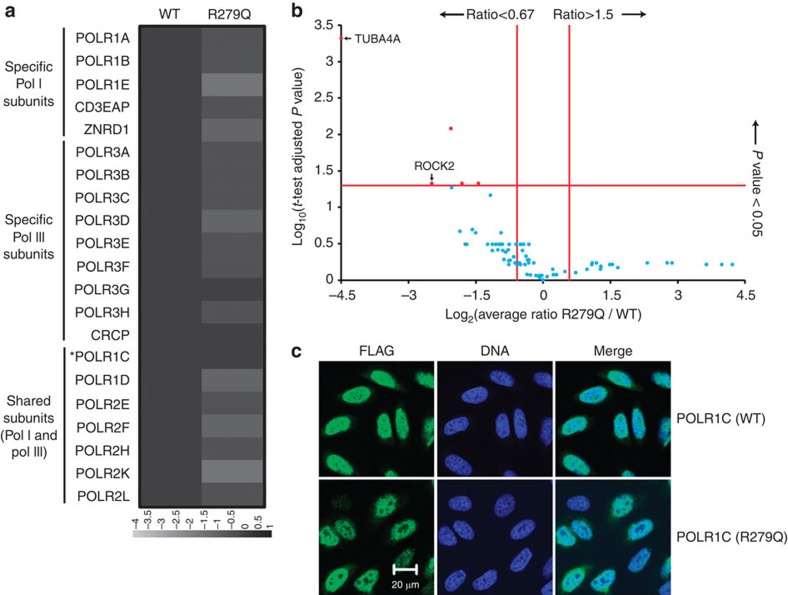
Impact of a TCS-causative mutation in *POLR1C* on polymerase assembly and cellular localization. FLAG-tagged POLR1C variants, either the wild type (1C) or a mutated version with the R279Q substitution, were expressed, affinity-purified and used in anti-FLAG immunofluorescence experiments as in Fig. 3. (**a**) Affinity purification coupled to mass spectrometry data is represented in the form of a heatmap that contains the log_2_-transformed average spectral count ratios R279Q/WT across all three replicates. Spectral counts were computed with Mascot (see legend to Fig. 3 for details). (**b**) Volcano plot of the log_2_-transformed average spectral count ratios N279Q/WT (*x* axis) and the –log_10_-transformed *P*-values (adjusted with the Benjamini–Hochberg procedure) resulting from the two-tailed one-sample *t*-tests of the high-confidence interactors of POLR1C. Red proteins show a level of differential interaction with POLR1C that is statistically significant, while blue proteins do not. Proteins with a log_2_-transformed average spectral count ratio <−4.5 were capped to −4.5 for display purposes. (**c**) Immunofluorescence data showing the cellular localization of tagged POLR1C variants are shown. Scale bar, 20 μm.

To investigate the potential pathogenic role of these mutations, we evaluated the impact of the two homozygous mutations (Table 1) on the function of nuclear POLR1 and POLR3. FLAG-tagged versions of the wild-type (WT) form of POLR1C and its variants having the p.Asn32Ile (N32I) or p.Asn74Ser (N74S) substitution were expressed in HeLa cells. Anti-FLAG affinity purification was performed on cell extracts and the purified proteins were analysed using shotgun proteomics. These experiments were performed in triplicate. The expression level of the various forms of POLR1C (that is, WT and mutants) were equivalent and comparable (see Supplementary Fig. 2), and protein expression levels were normalized by the expression level of the bait in each purification (Supplementary Table 6). The tagged WT POLR1C pulled down all subunits of both POLR1 and POLR3 (Fig. 3a), a finding that was expected since POLR1C is a shared subunit of both polymerases (see Fig. 3c for a schematic representation). Both tagged mutated POLR1C (N32I and N74S) pulled down amounts of POLR1-specific (POLR1A, 1B, 1E, CD3EAP, TWISTNB and ZNRD1) and POLR1/POLR3-shared subunits (POLR2E, 2F, 2H, 2K, 2L, 1C and 1D) that were not significantly different from those pulled down by the WT (this is especially true for the POLR1-specific subunits). However, both mutated subunits pulled down lower amounts of POLR3 (most specific subunits) relative to WT. This finding suggests that the mutations lead to a selective defect in POLR3 assembly, and not in POLR1. Indeed, assembly of nuclear RNA polymerases has previously been shown to occur in the cell's cytoplasm and defects in RNA polymerase assembly caused by functional disruption of the RNA Polymerase-Associated proteins was previously shown to lead to cytoplasmic accumulation of polymerase subunits^15^,^16^,^17^,^18^,^19^,^20^,^21^. Notably, the position of mutated residues in the proposed structure of POLR3 is compatible with defects in enzyme assembly and/or folding (see Supplementary Fig. 1). To further confirm that mutated POLR1C variants (N32I and N74S) are impaired in supporting enzyme assembly and nuclear import, immunofluorescence studies were performed using anti-FLAG antibodies. The results reveal an accumulation of both mutated POLR1C subunits, but not the WT subunit, in the cytoplasm (Fig. 3d). We then performed chromatin immunoprecipitation (ChIP) of FLAG-tagged POLR1C followed by high-throughput sequencing (ChIP-Seq), as a proxy of gene transcription activity^22^,^23^,^24^, to investigate the impact of *POLR1C* mutations on gene occupancy by POLR1 and POLR3. After alignment of the reads to the human reference genome (hg19), we compared occupancy of WT and mutated POLR1C variants over 659 POLR3-transcribed genes, including all transfer RNA (tRNA) and 5S ribosomal RNA genes (Supplementary Table 7). As expected, mutated POLR1C variants displayed reduced binding to POLR3-transcribed genes compared with WT POLR1C for all three classes of POLR3-transcribed genes (classified according to their regulatory elements; see legend to Fig. 4a). In contrast, there were no differences in WT and mutated POLR1C occupancy over the ribosomal RNA gene transcribed by POLR1 (Fig. 4c). Together, these results indicate that the N32I and N74S substitutions in the POLR1/POLR3-shared subunit POLR1C specifically interfere with assembly, nuclear import and chromatin association of POLR3. To compare the roles of leukodystrophy versus TCS-causing mutations in the biogenesis of POLR1 and POLR3, we expressed FLAG-tagged POLR1C with the p.Arg279Gln (R279Q) mutation in HeLa cells, affinity-purified the tagged subunit and identified the purified interactors using mass spectrometry. Contrary to POLR1C (N32I) and POLR1C (N74S), none of the subunits of POLR1 and POLR3 were pulled down by tagged POLR1C (R279Q) in amounts that were statistically significantly different from the WT (Fig. 5a,b, Supplementary Table 6), suggesting that this TCS-causing mutation does not affect the assembly of these polymerases. Notably, however, immunofluorescence results indicate that POLR1C (R279Q) targeting to the nucleolus is impaired as compared with the WT subunit (Fig. 5c) and the N32I- and N74S-mutated subunits (see Fig. 3d).

## Discussion

With the advent of exome sequencing, it is becoming increasingly apparent that allelic heterogeneity in genes encoding essential proteins, such as those involved in transcription, results in highly variable phenotypes. A previous report of mutations in *POLR1C* (ref. 14) highlighted the discovery of the first cases of TCS with an autosomal recessive mode of inheritance. TCS (MIM 154500, 613717, 248390) is characterized by an abnormal craniofacial development and is caused, from most to least frequent, by mutations in *TCOF1* (dominant), *POLR1D* (dominant) or *POLR1C* (recessive)^14^. TCS caused by mutations in *POLR1D* or *POLR1C* has been proposed to arise as a consequence of a decreased quantity of functional ribosomes in the neuroepithelium and the neuronal crest cells during critical points of embryogenesis^14^,^25^. We assessed the role of a TCS-causing mutation (R279Q) in the biogenesis of POLR1 and POLR3. Our results (see Fig. 5, Supplementary Table 6) indicate that this mutation does not impair polymerase assembly, as opposed to leukodystrophy-causing mutations (see Fig. 3), but affect targeting to the nucleolus, the site for Pol I transcription.

Our findings suggest that improper assembly and nuclear import of POLR3 resulting from leukodystrophy-causative mutations lead to decreased availability of the complex at the chromatin. As POLR3 binding is well correlated to tRNA expression^22^,^23^,^24^, this decreased POLR3 occupancy is likely to cause reduced transcription of tRNAs and other essential small non-coding RNAs. One hypothesis is that mutations in *POLR3A*, *POLR3B* or *POLR1C* lead to decreased levels of certain tRNAs crucial for the synthesis of proteins essential for central nervous system myelin development. tRNA function has also been suggested to be impaired in other white matter disorders^26^,^27^,^28^,^29^,^30^ caused by mutations in tRNA-aminoacyl synthetases, including hypomyelinating leukodystrophies such as *RARS*-associated hypomyelination^30^ and Hypomyelination with Brain Stem and spinal cord involvement and Leg spasticity^29^. Of note, tRNA synthetases have not been found in our POLR3 purifications, suggesting that tRNA aminoacylation is not coupled with POLR3 transcription. An alternative hypothesis would involve changes in the expression of other essential small non-coding RNAs synthesized by POLR3.

In conclusion, our sequencing study of 18 cases with compatible clinical and/or radiological features with 4H or POLR3-related leukodystrophy identified 13 different *POLR1C* mutations in eight cases. *POLR1C* joins an emerging group of genes with dual roles in pathogenesis of human diseases^31^, expanding the clinical phenotype associated with constitutional mutations in this gene and opening new aspects in the annotation and assessment of pathogenicity of sequence variants. Furthermore, our functional analyses on the mutational impact of *POLR1C* bring the first insights into the pathophysiology of POLR3-related disorders.

## Methods

### Patients and exome sequencing

Informed consent was obtained from all participants. The project was approved by the research ethics committee of the Montreal Children Hospital (11-105-PED), the institutional review board of the VU University Medical Center, Neuroscience Campus, Amsterdam, the Netherlands, the Children's National Health System as part of the Myelin Disorders Bioregistry Project in Washington, DC and the University of Queensland, Australia. Genomic DNA was extracted from peripheral blood leukocytes of patients and family members using the Qiagen Gentra Puregene Blood Kit (Qiagen, Hilden, Germany) according to the manufacturer's instructions. Exome sequencing was performed in two cases by PerkinElmer (Branford, Connecticut, USA) and in a third case by the Institute for Molecular Biology at the University of Queensland. Cases one and two were sequenced using PerkinElmer's sequencing service using the Agilent Sure Select Human All Exon Capture V4 Kit and exome sequencing for these two cases was performed (two paired-end 100-bp reads) with the Illumina HiSeq 2000 system. Reads were aligned to the reference human genome (UCSC Genome Browser hg19) with the Genome Analysis Toolkit (GATK)^32^,^33^, SAMtools^32^,^34^, Picard (see web resource) and CASAVA v1.8 (ref. 35) and annotated using the snpEff software tool (http://snpeff.sourceforge.net/), as well as visualization tools from PerkinElmer. Subsequent analyses were performed using the Ingenuity software package (Qiagen, Redwood City, USA). Exome enrichment for case three was performed using the Illumina Nextera Rapid Capture kit and sequenced on an Illumina HiSeq 2000 (2 × 100 bp paired-end). Reads were aligned to the reference human genome (GRCh37) and pedigree-informed variant calling was performed using the Real Time Genomics (RTG) integrated analysis tool rtg Family v3.2 (ref. 36). Variants were annotated using SnpEff v3.4 (ref. 37). In all cases, filtering queries were created as specific presets that allowed *in silico* reduction of variant lists down to candidates with correlation to phenotype, transmission mode of inheritance and alteration classification of pathogenicity. Sanger sequencing and co-segregation analysis were performed on genomic DNA using primer pairs designed with the primer3 software package and the genomic sequence of *POLR1C* (NM_203290; GRCh37/hg19). PCR products were forward- and reverse-sequenced at the McGill University and Genome Quebec Innovation Centre using an ABI 3730xl DNA Analyzer (ABI; Applied Biosystems, Foster City, CA, USA). Sequences were analysed using SeqMan 4.03 (DNAStar, Wisconsin, USA) and Chromas 1.62 (Technelysium Pty, Ltd, Australia).

### Immunofluorescence and western blot analyses

Transfection experiments for generating stable HeLa cell lines expressing FLAG-tagged versions of POLR1C variants used lipofectamine, as described by the supplier (Invitrogen, Carlsbad, CA, USA)^18^. The antibodies used in this study were obtained from the following sources: anti-FLAG monoclonal primary antibody (Sigma-Aldrich, St Louis, Missouri, USA), Alexa Fluor 488 fluorescence secondary antibody (Invitrogen) and anti-GAPDH (FL-335; Santa Cruz Biotechnology, Santa Cruz, CA, USA). Immunofluorescence studies used an anti-FLAG (dilution 1/300) antibody and the secondary antibody Alexa Fluor 488 (1/200) to localize exogenously expressed FLAG-POLR1C variants in HeLa cells. For western blot analysis, anti-FLAG (dilution 1/3,000) was used to detect the FLAG-POLR1C variants and anti-GAPDH (dilution 1/2,000;) for loading control^18^.

### Protein affinity purification coupled to mass spectrometry

Generation of cell lines stably expressing FLAG-tagged POLR1C subunits (WT and mutated) and affinity purification from the soluble fraction were performed using standard procedures^38^,^39^. The eluates were digested with trypsin and the resulting tryptic peptides were purified and identified with tandem mass spectrometry (LC-MS/MS) using a microcapillary reversed-phase high-pressure liquid chromatography-coupled LTQ-Orbitrap (ThermoElectron) quadrupole ion trap mass spectrometer with a nanospray interface, as recently described^40^. Protein database searching and protein spectral count quantification were performed with Mascot (version 2.3.02)^41^. The NCBI_Human protein sequence database was downloaded on 20 February 2014. Known protein contaminants such as keratins, which are not expressed in HeLa cells, were excluded from the data set. Undistinguishable protein isoforms were considered as a single protein. For each LC-MS/MS analysis, protein spectral counts were normalized by the spectral count of the FLAG-POLR1C in order to allow the comparison of different purifications. To simulate the background noise of the LC-MS/MS analysis of a given sample, spectral counts reported as 0 by Mascot were replaced by randomly generated spectral count values that are normally distributed with a mean and s.d. equal to those of the lowest 20% spectral count values from the LC-MS/MS analysis. Each replicate LC-MS/MS analysis of the affinity purifications of the FLAG-POLR1C mutants (MUT; N32I, N74S and R279Q) was paired with a LC-MS/MS analysis of the WT FLAG-POLR1C that was performed at the same time. The set of high-confidence interactors of POLR1C for a given mutant analysis was identified by comparing the spectral counts of the interactors obtained from the purifications of the paired WT POLR1C and the MUT POLR1C to those of the proteins purified with an empty vector (EV) of the FLAG tag (nonspecific interaction). A protein is labelled as a high-confidence interactor if it was identified and quantified in all three replicates of WT POLR1C or MUT POLR1C and that the ratio of the average spectral counts across the three replicates (WT/EV or MUT/EV) was greater than 5. These stringent criteria allow us to eliminate the vast majority of nonspecific interactors of POLR1C for the analysis of the interactions of each mutant. For each high-confidence interacting protein, a two-tailed one-sample *t*-test was performed on the spectral count ratios (MUT/WT). The resulting *P* values were adjusted for multiple hypothesis testing using the Benjamini–Hochberg procedure^42^. To maximize the specificity of our approach, a protein is deemed to show a level of differential interaction with POLR1C that is statistically significant when its adjusted *P* value is <0.05 and that its average spectral count fold-change (MUT/WT) is <0.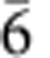 or >1.5.

### ChIP-sequencing and data analysis

Stable HeLa cell lines expressing FLAG-tagged POLR1C (WT or mutated) were cultured to 80% confluence and crosslinked with 1% formaldehyde directly in the cell medium for 5 min followed by a 5-min quenching in 125 mM glycine. For ChIP experiments^43^, nuclei from 3 × 10^6^ cells were lysed and re-suspended in sonication buffer (10 mM Tris-HCl pH 8, 140 mM NaCl, 1 mM EDTA, 0.5 mM EGTA, 0.5% Triton, 0.5% SDS and protease inhibitors). Chromatin was prepared by sonicating the nuclei in a Covaris E-Series E22 for 6 min at Duty 2. This generated chromatin fragments of 500 bp on average. Sonicated chromatin was immunoprecipitated using 25 μl of anti-FLAG M2 Magnetic beads (Sigma) for 4 h. The beads were then washed and eluted, followed by phenol:chloroform-isoamyl (25:24:1 pH 8, Invitrogen) extraction and ethanol precipitation. Sequencing libraries were prepared from input and ChIP eluates (WT and mutated) using the TruSeq DNA library preparation kit (Illumina). Libraries were sequenced on an Illumina HiSeq 2000 (2 × 50 cycles paired-end). Quality control of the sequencing data was performed with FastQC and low-quality bases and adapter sequences were trimmed with Trimmomatic^44^. Unique reads were aligned to the reference human genome (build hg19) or to the human rDNA reference sequence (rDNA; NCBI accession number: HSU13369) with Bowtie version 2.1.0 (ref. 45). Input DNA signal was subtracted from ChIP signal. POLR1C peaks were called using MACS version 2.0.10 (ref. 46). Peaks were annotated with HOMER version 4.5.0 (ref. 47) using GENCODE Genes V19 annotations^48^ and the tRNA Genes track of the UCSC Genome Browser^49^,^50^. Data visualization was performed with the Integrative Genomics Viewer^51^. To compare chromatin binding between WT and mutated POLR1C variants over all POLR3-transcribed genes divided into three classes according to their regulatory elements (see legend to Fig. 4), we used the annotation mode of the Versatile Aggregate Profiler with the transcription start site as the only reference point and 100 windows of 10 base pairs each on both sides of the reference point^52^.

## Additional information

**Accession codes**: The high-throughput sequencing data generated in this study have been deposited into the NCBI Sequence Read Archive under the accession code SRP057978.

**How to cite this article**: Thiffault, I. *et al*. Recessive mutations in *POLR1C* cause a leukodystrophy by impairing biogenesis of RNA polymerase III. *Nat. Commun.* 6:7623 doi: 10.1038/ncomms8623 (2015).

## Supplementary Material

Supplementary Information Supplementary Figures 1-2 and Supplementary Tables 1-7

## Figures and Tables

**Table 1:** Major clinical and MRI findings in index patients with mutations in *POLR1C*.
