# Supplementary material for: Recessive mutations in POLR1C cause a leukodystrophy by impairing biogenesis of RNA polymerase III
Source: Nat Commun. 2015 Jul 7;6:7623. doi: 10.1038/ncomms8623 (PMC4506509; doi:10.1038/ncomms8623)
Supplement: Supplementary Information — Supplementary Figures 1-2 and Supplementary Tables 1-7 [file ncomms8623-s1.pdf]

Supplementary Figure 1

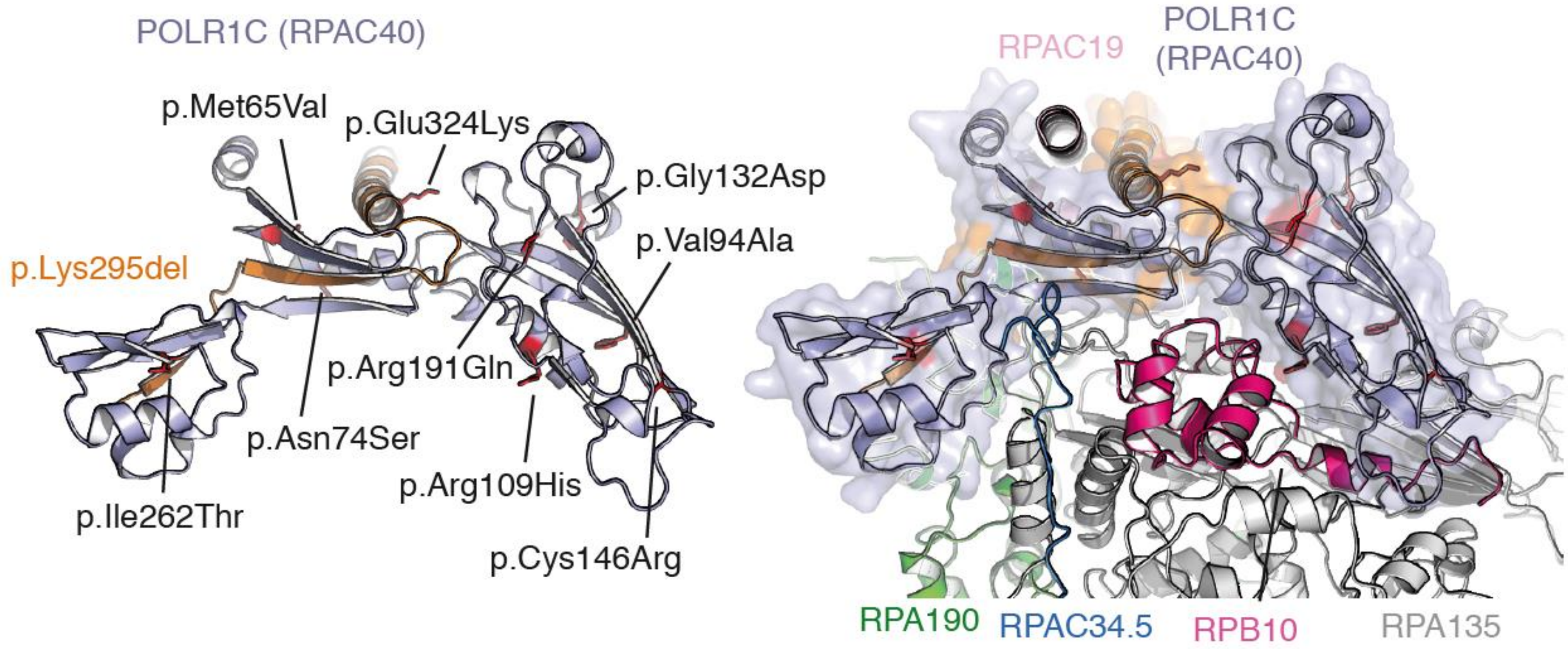

3-dimensional representation of POLR1C extrapolated from the yeast Pol I structure. *POLR1C* mutations affect amino acids that are conserved in *S. cerevisiae*. Due to their location at the hydrophobic core of the subunit, four of the mutations (p.Met65Val (Leu56), p.Val94Ala (Phe85), p.Ile262Thr (Ile256), p.Glu324Lys (K316)) likely impair, at least partly, the overall folding of POLR1C. The location of p.Arg109His (Arg100) in the vicinity of POLR2L (RPB10) can be extrapolated to a similar location in Pol III and suggests that the interaction of POLR1C to the remainder of the Pol III could be weakened. As p.Asn74Ser (Asn65) is located at the interface with POLR1B (RPA135), a similar conclusion can be drawn regarding the interaction with POLR3B. The mutation p.Cys146Arg (Cys140) are predicted to impair the overall folding of the subunit, altering its stability. The deletion p.Lys295del (Asp287) is represented on the left panel while the deleted portion of the protein is shown in light grey on the right panel. p.Thr26Ile, p.Asn32Ile and p.Lys154Rfs*4 were not represented since they are not found in the POLR1C structure model or represent a large truncation of the protein.

Supplementary Figure 2

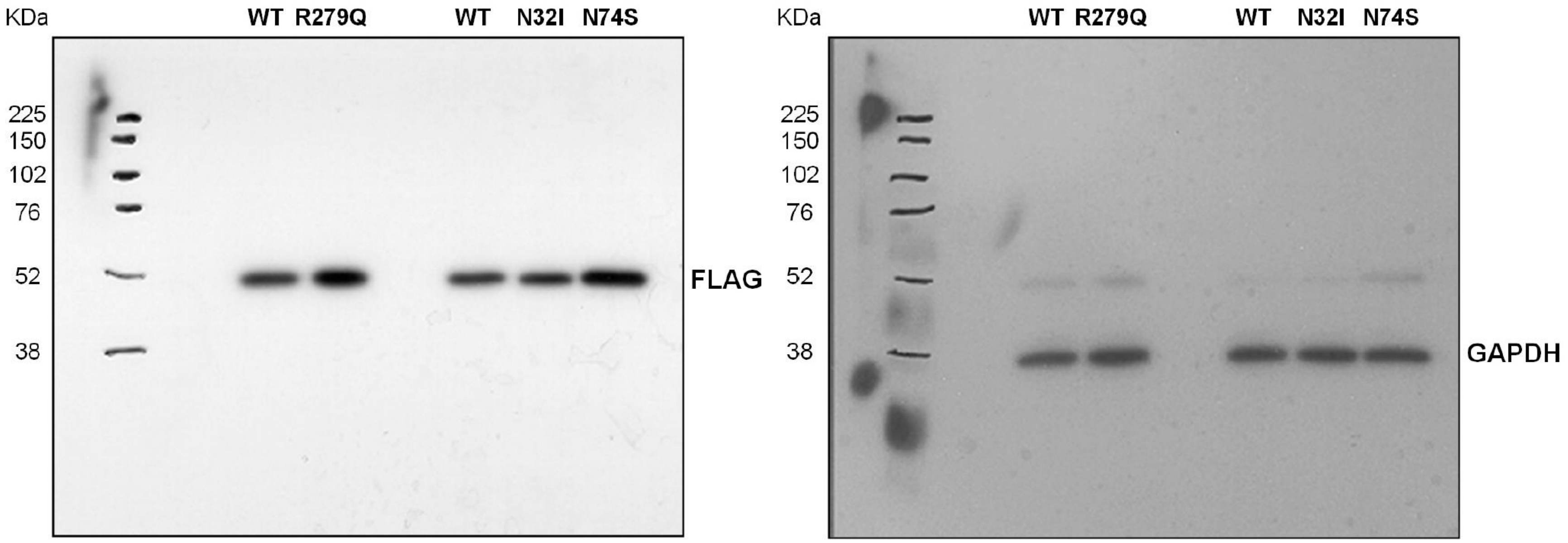

Full scan of the Western blot showing expression levels of FLAG-tagged POLR1C variants (WT and mutants) using GAPDH as a loading control.

Supplementary Table 1: Exome sequencing summary

NGS summary for each sample	Patient 1	Patient 2	Patient 3
Total captured (G)	4.9	6.1	3.7
% of captured with > 10 (%)			95.8
Average coverage of captured region	56.06x	58.42x	104.8x
Total number of SNPs	73,126	70,768	35,667
Total number of INDELs	6,662	6,598	2941
Homozygous			
NS/I	4,851	4,624	4,809
*Novel	27	8	22
Common on both samples	0	0	0
Different mutations in same gene	1	1	0
Heterozygous			
NS/I	7,103	6,837	5,633
*Novel	238	180	351
Common on both samples	12	18	11
Common to ALL samples	1	1	1
# X-linked	1,186	1,160	1,030

***Novel variants refer to those not present in the dbSNP and 1000 Genome databases**

Legend: NS: non-synonymous; I: indel

Supplementary Table 2a: *POLR1C* mutations

Patient #	Family #	WES	Exon	Chromosome position	cDNA mutation	Protein alteration	Parental origin
1	I	By WES	2	chr6:43485069	c.95A>T	p.Asn32Ile	PATERNAL
			2		c.95A>T	p.Asn32Ile	MATERNAL
2	II	By WES	3	chr6:43487150	c.221A>G	p.Asn74Ser	PATERNAL
			3		c.221A>G	p.Asn74Ser	MATERNAL
3	III	By WES	5	chr6:43487857	c.436T>C	p.Cys146Arg	MATERNAL
			8	chr6:43488747-43488749	c.883_885delAAG	p.Lys295del	PATERNAL
4	IV	n.a	2	chr6:43485051	c.77C>T	p.Thr26Ile	PATERNAL
			4	chr6:43487520	c.326G>A	p.Arg109His	MATERNAL
5	V	n.a	3	chr6:43487122	c.193A>G	p.Met65Val	n.a
			6	chr6:43488082	c.572G>A	p.Arg191Gln	n.a
6	VI	n.a	4	chr6:43487520	c.326G>A	p.Arg109His	PATERNAL
			9	chr6:43488967	c.970G>A	p.Glu324Lys	MATERNAL
7	VII	n.a	5	chr6:43487816	c.395G>A	p.Gly132Asp	MATERNAL
			5	chr6:43487882-43487883	c.461_462delAA	p.Lys154Argfs*4	PATERNAL
8	VIII	n.a	4	chr6:43487475	c.281T>C	p.Val94Ala	PATERNAL
			7	chr6:43488492	c.785T>C	p.Ile262Thr	MATERNAL

Supplementary Table 2b: *POLR1C* minor allele frequency (MAF) from common databases

Patient #	Family #	cDNA mutation	Protein alteration	dbSNP	MAF	CEPH frequency	ExAC	EVS	dbSNP	HapMap	1000 Genome
1	I	c.95A>T	p.Asn32Ile			0%	n.a	n.a	n.a	n.a	n.a
		c.95A>T	p.Asn32Ile			0%					
2	II	c.221A>G	p.Asn74Ser			0%	0.00002442	0.0077	n.a	n.a	n.a
		c.221A>G	p.Asn74Ser			0%					
3	III	c.436T>C	p.Cys146Arg			0%	n.a	n.a	n.a	n.a	n.a
		c.883_885delAAG	p.Lys295del			0%	n.a	n.a	n.a	n.a	n.a
4	IV	c.77C>T	p.Thr26Ile			0%	n.a	n.a	n.a	n.a	n.a
		c.326G>A	p.Arg109His			0%	n.a	n.a	n.a	n.a	n.a
5	V	c.193A>G	p.Met65Val			0%	0.0008546	n.a	G=0.000/1	n.a	n.a
		c.572G>A	p.Arg191Gln			0%	0.00007328	0.0231	n.a	n.a	n.a
6	VI	c.326G>A	p.Arg109His			0%	n.a	n.a	n.a	n.a	n.a
		c.970G>A	p.Glu324Lys			0%	n.a	n.a	n.a	n.a	n.a
7	VII	c.395G>A	p.Gly132Asp	rs201320592	0.15%	0%	0.0003528	n.a	n.a	n.a	n.a
		c.461_462delAA	p.Lys154Argfs*4			0%	0.000008133	n.a	n.a	n.a	n.a
8	VIII	c.281T>C	p.Val94Ala			0%	n.a	0.0384	A=0.000/2	n.a	n.a
		c.785T>C	p.Ile262Thr			0%	0.00002441	n.a	n.a	n.a	n.a

Supplementary Table 2c: *In silico* analysis of *POLR1C* variant pathogenicity

Patient #	Family #	cDNA mutation	Protein alteration	MutationTASTER	SIFT	POLYPHEN2	PROVEAN
1	I	c.95A>T	p.Asn32Ile	DISEASE-CAUSING [0.99]	TOLERATED [0.158]	DAMAGING [0.93]	DELETERIOUS [3.651]
		c.95A>T	p.Asn32Ile				
2	II	c.221A>G	p.Asn74Ser	DISEASE-CAUSING [0.99]	DAMAGING [0.002]	DAMAGING [0.99]	DELETERIOUS [-4.869]
		c.221A>G	p.Asn74Ser				
3	III	c.436T>C	p.Cys146Arg	DISEASE-CAUSING [0.99]	DAMAGING [0.00]	DAMAGING [0.99]	DELETERIOUS [-10.947]
		c.883_885delAAG	p.Lys295del	n.a	n.a	n.a	DELETERIOUS [-8.007]
4	IV	c.77C>T	p.Thr26Ile	DISEASE-CAUSING [0.99]	DAMAGING [0.002]	DAMAGING [0.79]	NEUTRAL [-2.489]
		c.326G>A	p.Arg109His	DISEASE-CAUSING [1.00]	DAMAGING [0.00]	DAMAGING [1.00]	DELETERIOUS [-4.895]
5	V	c.193A>G	p.Met65Val	DISEASE-CAUSING [0.99]	DELETERIOUS [0.03]	BENIGN	DELETERIOUS [-4.869]
		c.572G>A	p.Arg191Gln	DISEASE-CAUSING [0.99]	TOLERATED [0.52]	DAMAGING [0.96]	NEUTRAL [-1.339]
6	VI	c.326G>A	p.Arg109His	DISEASE-CAUSING [1.00]	DAMAGING [0.00]	DAMAGING [1.00]	DELETERIOUS [-4.895]
		c.970G>A	p.Glu324Lys	DISEASE-CAUSING [1.00]	TOLERATED [0.292]	DAMAGING [0.999]	DELETERIOUS [-2.927]
7	VII	c.395G>A	p.Gly132Asp	DISEASE-CAUSING [1.00]	TOLERATED [0.19]	DAMAGING [0.98]	NEUTRAL [-1.93]
		c.461_462delAA	p.Lys154Argfs*4	n.a	n.a	n.a	n.a
8	VIII	c.281T>C	p.Val94Ala	DISEASE-CAUSING [1.00]	DAMAGING [0.00]	DAMAGING [0.695]	DELETERIOUS [-3.549]
		c.785T>C	p.Ile262Thr	DISEASE-CAUSING [1.00]	DAMAGING [0.00]	DAMAGING [0.983]	DELETERIOUS [-4.145]

Supplementary Table 2d: *POLR1C S. cerevisiae* data

Patient #	Family #	cDNA mutation	Protein alteration	Variant in *S. cerevisiae*	Impact on S. cerevisiae
1	I	c.95A>T	p.Asn32Ile	Phe23	Not seen in the model of POLR1C
		c.95A>T	p.Asn32Ile		
2	II	c.221A>G	p.Asn74Ser	Asn65	At interface POLR1B (A135)
		c.221A>G	p.Asn74Ser		
3	III	c.436T>C	p.Cys146Arg	Cys140	Impairs overall folding of the subunit
		c.883_885delAAG	p.Lys295del	Asp287	Impairs overall folding of the subunit
4	IV	c.77C>T	p.Thr26Ile	Ser17	Not present in POLR1C structural model
		c.326G>A	p.Arg109His	Arg100	At the interface with RPB10
5	V	c.193A>G	p.Met65Val	Leu56	Hydrophobic core, close to RPAC19
		c.572G>A	p.Arg191Gln	Val185	Exposed to the solvent
6	VI	c.326G>A	p.Arg109His	Arg100	At the interface with RPB10
		c.970G>A	p.Glu324Lys	Lys316	Stabilizing subunit itself
7	VII	c.395G>A	p.Gly132Asp	Phe126	Impairs overall folding of the subunit
		c.461_462delAA	p.Lys154Argfs*4	Lys148del	Impairs overall folding of the subunit
8	VIII	c.281T>C	p.Val94Ala	Phe 85	Impairs overall folding of the subunit
		c.785T>C	p.Ile262Thr	Ile256	Impairs overall folding of the subunit

Supplementary Table 3: Primers covering all exons and intron-exon boundaries of *POLR1C* gene

Exon	Primers Sequences	Tm (°C)	Length of products (bp)
POLR1C exons 01-02 Forward	ccggaacacaaaaaggagaa	60.08	600
POLR1C exons 01-02 Reverse	aaacggagcgtctagcacat	59.9	
POLR1C exon 03 Forward	gcaaagggaggtttttggat	60.3	445
POLR1C exon 03 Reverse	ccctacctatccccgtaacc	59.55	
POLR1C exon 04 Forward	tggaattttgctgagcatttt	59.71	422
POLR1C exon 04 Reverse	gggagacaaggcaagtgttc	59.7	
POLR1C exons 05-06 Forward	ttgccttgtctcccaagttt	59.71	597
POLR1C exons 05-06 Reverse	cagcaaaagcacaaccctct	60.43	
POLR1C exons 07-08 Forward	agaaagccaagagggttgtg	59.33	662
POLR1C exons 07-08 Reverse	gactggggaccaaagagaca	60.09	
POLR1C exon 09 Forward	aagaaggttgtgaggcttgc	59.48	638
POLR1C exon 09 Reverse	cctcagcctcctgagaattg	59.94	

Supplementary Table 4a: Demographic data of index cases with *POLR1C* mutations

Patient #	Family #	Age in 2014 (years)	Gender	Ethnicity (country of origin)	Consanguinity	Age at last assessment (years)	Age of onset (years)
1	I	9	M	Libyan	Yes	8	0.5
2	II	10	M	Hungarian	No	10	1
3	III	5	M	Asian (China)	No	4	1
4	IV	8	F	Caucasian (Armenia/Russia)	No	6	2.5
5	V	10	F	Caucasian	No	9	1.5
6	VI	20	F	Caucasian (Turkey)	Suspected	18	4
7	VII	34	M	Caucasian	No	33	2
8	VIII	3	F	Caucasian	No	2	1

Supplementary Table 4b: Clinical data (neurological) of index cases with *POLR1C* mutations

Patient #	Family #	Symptoms at onset	Developmental delay	Walking without support (age in months)	Abnormal cognition	Upper motor neuron signs	Tremor	Cerebellar findings	Age at wheelchair (years)
1	I	Delayed motor development	+	22	+	+	+	+	3
2	II	Ataxia, tremor, head titubation	+	18	+	+	+	+	9
3	III	Delayed motor development and failure to thrive	+	Never independently	+	+	+	+	Always
4	IV	Tremor, dysmetria	+	15	-	+	+	+	-
5	V	Delayed motor development	+	24	+	+	+	+	Always
6	VI	Clumsy gait, frequent falls	-	18	+	+	+	+	9 (long distances)
7	VII	Delayed motor development	+	Never independently	+	-	+ (with prominent myoclonus)	+	Puberty
8	VIII	Delayed motor development	+	24 (with support)	-	-	+	+	-

Supplementary Table 4c: Clinical data (non-neurological) of index cases with *POLR1C* mutations.

Patient #	Family #	Deteriorations with infections	Myopia	Dental abnormality	Hypogonadotropic hypogonadism	Clinical features suggestive of Treacher Collins syndrome*
1	I	-	-	+	Too young	-
2	II	+	-	-	Too young	-
3	III	-	-	-	Too young	-
4	IV	+	-	+	Too young	-
5	V	+	+	-	Too young	-
6	VI	+	+	-	-	-
7	VII	-	+	-	-	-
8	VIII	-	-	+	Too young	-

Supplementary Table 5: MRI findings of index cases with *POLR1C* mutations

Patient #	Family #	Gender	Age at last good quality MRI (years)	Hypo-myelination	Myelination of optic radiation	Hypointense part of CI	Thin corpus callosum	Hypointense lateral thalami	Relatively hypointense pallidum	Supratentorial atrophy	Cerebellar atrophy	Hypointense dentatus (preserved myelination)
1	I	M	6	+	+	-	+*	+	+	+*	-	+
2	II	M	4.5	+	+	+	+*	+	-	-	-	-
3	III	M	4.5	+#	+	+	+	+	-	-	-	+
4	IV	F	6	+	+	-	+*	+	-	-	+*	+
5	V	F	8	+	+	-	+*	+	-	-	++*	+
6	VI	F	18	+	+	+	+++*	+	-	-	++*	+
7	VII	M	33	+	+	+	+++*	+	+	++*	+++*	-
8	VIII	F	2.75	+	+	+	+*	+	-	-	++*	-

Legend:

M: Male; F: Female

–: absent; +: present

+*: mild; ++*: moderate; +++*: severe; +#: over time developed some rarefaction of affected white matter

Supplementary Table 6a: Quantification of the high-confidence interactors of the N32I mutant POLR1C against WT POLR1C.

Protein Symbol	Quantification Ratio 1	Quantification Ratio 2	Quantification Ratio 3	Average Ratio	Two-tailed one-sample t-test p-value	BH-Adjusted p-value	Log2 (Average Ratio)	(-1)LOG10(BH-Adjusted p-value)
POLR3E	0.569	0.553	0.571	0.564	1.69E-04	1.25E-02	-0.826	1.904
POLR3B	0.525	0.564	0.550	0.546	6.37E-04	2.36E-02	-0.872	1.628
POLR3G	0.154	0.065	0.196	0.138	1.99E-03	4.27E-02	-2.853	1.369
POLR3A	0.271	0.216	0.125	0.204	2.88E-03	4.27E-02	-2.294	1.369
POLR1D	0.496	0.480	0.402	0.459	2.89E-03	4.27E-02	-1.122	1.369
POLR3F	0.237	0.106	0.258	0.200	3.54E-03	4.37E-02	-2.320	1.360
POLR3C	0.357	0.135	0.214	0.235	7.17E-03	7.58E-02	-2.086	1.120
POLR2L	0.604	0.436	0.560	0.534	1.14E-02	1.05E-01	-0.906	0.978
MAF1	0.423	0.145	0.448	0.339	2.08E-02	1.68E-01	-1.561	0.775
POLR3H	0.470	0.156	0.093	0.239	2.27E-02	1.68E-01	-2.062	0.775
CRCP	0.141	0.069	0.576	0.262	4.32E-02	2.90E-01	-1.932	0.537
POLR2F	0.585	0.785	0.493	0.621	4.82E-02	2.97E-01	-0.687	0.527
RPL32	0.169	0.748	0.448	0.455	8.26E-02	4.70E-01	-1.136	0.328
TUBA4A	0.014	0.751	0.008	0.258	9.50E-02	5.02E-01	-1.957	0.299
POLR2K	0.423	0.655	0.874	0.650	1.15E-01	5.57E-01	-0.620	0.254
DDX21	1.409	1.309	2.017	1.578	1.20E-01	5.57E-01	0.659	0.254
GPN1	1.208	2.127	1.614	1.650	1.35E-01	5.73E-01	0.722	0.242
POLR1E	2.114	1.651	1.177	1.647	1.39E-01	5.73E-01	0.720	0.242
RSL1D1	1.076	1.964	2.017	1.686	1.54E-01	5.96E-01	0.753	0.224
RPL10	0.242	0.982	0.336	0.520	1.75E-01	5.96E-01	-0.944	0.224
POLR2H	0.458	0.982	0.336	0.592	1.76E-01	5.96E-01	-0.756	0.224
RPL3	0.625	0.890	0.144	0.553	1.77E-01	5.96E-01	-0.854	0.224
RPL36	0.282	0.748	0.896	0.642	1.93E-01	6.21E-01	-0.639	0.207
RPL27A, RPL27AP6	0.528	1.018	0.288	0.612	2.12E-01	6.55E-01	-0.709	0.184
GPN3	3.382	2.291	0.934	2.202	2.32E-01	6.86E-01	1.139	0.164
RPL13	0.497	1.078	0.526	0.701	2.54E-01	7.14E-01	-0.513	0.146
NONO	1.424	3.927	1.345	2.232	2.83E-01	7.14E-01	1.158	0.146
RPL10A	1.691	1.428	0.896	1.339	2.85E-01	7.14E-01	0.421	0.146
RPL27	1.127	0.327	0.538	0.664	2.96E-01	7.14E-01	-0.590	0.146
POLR3D	0.141	0.620	1.103	0.621	3.06E-01	7.14E-01	-0.687	0.146
LIMCH1	1.550	5.236	1.345	2.710	3.09E-01	7.14E-01	1.439	0.146
HIST1H3A, HIST1H3B, HIST1H3I	4.358	2.618	0.523	2.500	3.09E-01	7.14E-01	1.322	0.146
HIST1H1B	1.344	1.964	0.874	1.394	3.38E-01	7.37E-01	0.479	0.133
LOC728026, PTMA, PTMAP2	2.325	6.545	0.672	3.181	3.39E-01	7.37E-01	1.670	0.133
POLR2E	0.370	1.212	0.518	0.700	3.67E-01	7.77E-01	-0.514	0.110

Protein Symbol	Quantification Ratio 1	Quantification Ratio 2	Quantification Ratio 3	Average Ratio	Two-tailed one-sample t-test p-value	BH-Adjusted p-value	Log2 (Average Ratio)	(-1)LOG10(BH-Adjusted p-value)
PLEC	1.205	0.655	0.336	0.732	4.01E-01	8.25E-01	-0.451	0.084
RPL26	7.610	1.496	0.465	3.191	4.29E-01	8.44E-01	1.674	0.074
RPL15	0.995	24.873	0.428	8.765	4.37E-01	8.44E-01	3.132	0.074
SUPT16H	1.190	0.655	0.672	0.839	4.55E-01	8.44E-01	-0.254	0.074
DHX9	1.283	0.655	0.336	0.758	4.76E-01	8.44E-01	-0.400	0.074
RPS9	0.935	1.122	0.303	0.786	4.80E-01	8.44E-01	-0.347	0.074
KPNA2	5.708	1.091	0.395	2.398	4.90E-01	8.44E-01	1.262	0.074
CAD	5.074	0.982	0.560	2.205	4.91E-01	8.44E-01	1.141	0.074
UBR5	0.846	2.618	0.864	1.443	5.30E-01	8.69E-01	0.529	0.061
RPL21P28	0.423	1.309	0.672	0.801	5.30E-01	8.69E-01	-0.319	0.061
RPL23A	1.268	0.842	0.224	0.778	5.40E-01	8.69E-01	-0.362	0.061
RPL4	0.846	1.275	0.312	0.811	5.67E-01	8.92E-01	-0.303	0.049
NOLC1	1.691	1.145	0.702	1.179	5.95E-01	8.97E-01	0.238	0.047
RPL28	0.705	1.440	0.168	0.771	5.98E-01	8.97E-01	-0.375	0.047
RPL8	0.664	1.386	0.448	0.833	6.15E-01	8.97E-01	-0.264	0.047
CD3EAP	1.127	1.428	0.773	1.110	6.21E-01	8.97E-01	0.150	0.047
PRPF8	3.160	0.873	0.403	1.479	6.31E-01	8.97E-01	0.564	0.047
RPLP0	1.153	1.078	0.374	0.868	6.49E-01	9.06E-01	-0.204	0.043
POLR2B	3.382	0.327	0.598	1.436	6.99E-01	9.58E-01	0.522	0.019
POLR1A	1.059	1.483	0.731	1.091	7.17E-01	9.59E-01	0.125	0.018
BAG2	1.015	1.309	0.336	0.887	7.32E-01	9.59E-01	-0.174	0.018
XRCC5	3.305	0.655	0.122	1.361	7.49E-01	9.59E-01	0.444	0.018
RPL21	0.846	1.309	0.672	0.942	7.90E-01	9.59E-01	-0.086	0.018
RUVBL1	0.789	0.727	1.793	1.103	7.93E-01	9.59E-01	0.142	0.018
HNRNPL	1.997	1.309	0.168	1.158	7.95E-01	9.59E-01	0.212	0.018
RPL7A	1.496	1.434	0.364	1.098	8.15E-01	9.59E-01	0.135	0.018
RPL13A	0.423	1.833	0.384	0.880	8.25E-01	9.59E-01	-0.185	0.018
RPS6	2.537	0.800	0.144	1.160	8.43E-01	9.59E-01	0.214	0.018
TCOF1	0.719	0.796	1.671	1.062	8.59E-01	9.59E-01	0.086	0.018
RPL6	0.689	1.595	0.526	0.937	8.67E-01	9.59E-01	-0.094	0.018
RPL34	0.987	1.309	0.588	0.961	8.70E-01	9.59E-01	-0.057	0.018
RPL14	0.940	1.484	0.403	0.942	8.70E-01	9.59E-01	-0.086	0.018
HNRNPF	2.086	0.561	0.061	0.903	8.88E-01	9.59E-01	-0.148	0.018
POLR1B	0.888	1.387	0.644	0.973	9.13E-01	9.59E-01	-0.040	0.018
RPL35A	1.691	0.935	0.244	0.957	9.27E-01	9.59E-01	-0.064	0.018
ZNRD1	0.846	1.174	0.952	0.991	9.31E-01	9.59E-01	-0.014	0.018
RPL18A	1.480	0.916	0.672	1.023	9.33E-01	9.59E-01	0.033	0.018
RPL7	0.669	2.014	0.398	1.027	9.61E-01	9.75E-01	0.039	0.011
RPL18	0.676	1.590	0.756	1.007	9.82E-01	9.82E-01	0.011	0.008
POLR1C	1.000	1.000	1.000	1.000	N/A	N/A	0.000	N/A

Supplementary Table 6b: Quantification of the high-confidence interactors of the N74S mutant POLR1C against WT POLR1C.

Protein Symbol	Quantification Ratio 1	Quantification Ratio 2	Quantification Ratio 3	Average Ratio	Two-tailed one-sample t-test p-value	BH-Adjusted p-value	Log2 (Average Ratio)	(-1)LOG10(BH-Adjusted p-value)
MAF1	0.031	0.087	0.067	0.062	3.06E-04	1.55E-02	-4.017	1.809
POLR3E	0.114	0.182	0.156	0.151	5.39E-04	1.55E-02	-2.729	1.809
POLR3A	0.129	0.203	0.212	0.181	1.04E-03	1.55E-02	-2.464	1.809
POLR3F	0.083	0.139	0.188	0.137	1.24E-03	1.55E-02	-2.869	1.809
POLR3H	0.094	0.205	0.112	0.137	1.59E-03	1.55E-02	-2.869	1.809
POLR2B	0.085	0.196	0.090	0.124	1.69E-03	1.55E-02	-3.017	1.809
CRCP	0.081	0.165	0.202	0.149	1.78E-03	1.55E-02	-2.744	1.809
RPL3	0.265	0.376	0.318	0.319	2.20E-03	1.66E-02	-1.646	1.779
RPL35A	0.085	0.224	0.074	0.127	3.04E-03	1.66E-02	-2.972	1.779
POLR3D	0.036	0.206	0.097	0.113	3.11E-03	1.66E-02	-3.143	1.779
RPL10	0.341	0.293	0.202	0.279	3.15E-03	1.66E-02	-1.843	1.779
GPN1	0.043	0.196	0.027	0.088	3.47E-03	1.66E-02	-3.500	1.779
POLR3G	0.095	0.235	0.067	0.133	3.54E-03	1.66E-02	-2.916	1.779
RPL27	0.341	0.293	0.162	0.265	5.26E-03	2.29E-02	-1.914	1.640
POLR3C	0.083	0.276	0.251	0.203	5.75E-03	2.34E-02	-2.297	1.631
TCOF1	0.295	0.223	0.441	0.320	8.84E-03	3.37E-02	-1.646	1.472
RPL4	0.192	0.432	0.260	0.295	1.02E-02	3.65E-02	-1.763	1.438
RPL14	0.227	0.470	0.378	0.358	1.19E-02	4.04E-02	-1.482	1.394
RUVBL1	0.043	0.377	0.090	0.170	1.55E-02	4.96E-02	-2.558	1.304
POLR3B	0.480	0.193	0.185	0.286	1.80E-02	5.14E-02	-1.805	1.289
POLR2F	0.085	0.313	0.432	0.277	1.92E-02	5.14E-02	-1.854	1.289
RPL13	0.215	0.506	0.211	0.311	1.95E-02	5.14E-02	-1.686	1.289
POLR2E	0.210	0.522	0.287	0.339	1.96E-02	5.14E-02	-1.559	1.289
URI1	0.038	0.046	0.405	0.163	2.02E-02	5.14E-02	-2.619	1.289
POLR2H	0.234	0.587	0.303	0.375	2.85E-02	6.66E-02	-1.416	1.176
POLR2L	0.151	0.522	0.135	0.269	2.86E-02	6.66E-02	-1.893	1.176
RPS6	0.182	0.478	0.520	0.393	2.95E-02	6.66E-02	-1.346	1.176
CCT7P2	0.114	0.196	0.540	0.283	3.15E-02	6.87E-02	-1.822	1.163
RPS9	0.076	0.335	0.526	0.312	3.42E-02	7.19E-02	-1.679	1.143
RPL21, RPL21P28	0.341	0.078	0.540	0.319	3.64E-02	7.41E-02	-1.646	1.130
RPLP0	0.210	0.598	0.495	0.434	3.97E-02	7.81E-02	-1.204	1.107
RPL8	0.128	0.598	0.378	0.368	4.33E-02	8.17E-02	-1.442	1.088
RPL7A	0.080	0.522	0.472	0.358	4.42E-02	8.17E-02	-1.482	1.088
RPL6	0.198	0.587	0.598	0.461	5.48E-02	9.33E-02	-1.117	1.030
RPL13A	0.068	0.626	0.116	0.270	5.50E-02	9.33E-02	-1.889	1.030
POLR1A	0.409	0.750	0.636	0.598	5.71E-02	9.33E-02	-0.741	1.030
CD3EAP	0.315	0.740	0.459	0.505	5.80E-02	9.33E-02	-0.987	1.030

Protein Symbol	Quantification Ratio 1	Quantification Ratio 2	Quantification Ratio 3	Average Ratio	Two-tailed one-sample t-test p-value	BH-Adjusted p-value	Log2 (Average Ratio)	(-1)LOG10(BH-Adjusted p-value)
RPL34	0.389	0.685	0.708	0.594	5.84E-02	9.33E-02	-0.751	1.030
RPL10A	0.681	0.142	0.270	0.364	5.96E-02	9.33E-02	-1.456	1.030
RPL7	0.236	0.722	0.270	0.409	6.38E-02	9.57E-02	-1.289	1.019
POLR1B	0.379	0.783	0.453	0.538	6.51E-02	9.57E-02	-0.893	1.019
TUBA4A	0.008	0.641	0.010	0.220	6.59E-02	9.57E-02	-2.186	1.019
RPL28	0.146	0.470	0.708	0.441	7.55E-02	1.07E-01	-1.180	0.970
POLR2K	0.272	0.783	0.324	0.460	7.95E-02	1.10E-01	-1.122	0.958
RPL27A, RPL27AP6	0.114	0.609	0.694	0.472	1.00E-01	1.36E-01	-1.083	0.868
WDR92	0.068	0.060	0.809	0.313	1.10E-01	1.45E-01	-1.678	0.839
RPL18	0.155	0.671	0.708	0.511	1.12E-01	1.45E-01	-0.968	0.839
RPL18A	0.298	0.704	0.809	0.604	1.26E-01	1.60E-01	-0.728	0.795
POLR1E	0.657	0.749	0.961	0.789	1.44E-01	1.79E-01	-0.342	0.747
RPL26	0.043	0.671	0.747	0.487	1.48E-01	1.81E-01	-1.039	0.743
RPL23A	0.043	0.671	0.809	0.508	1.72E-01	2.06E-01	-0.978	0.686
POLR1D	1.068	0.522	0.417	0.669	2.43E-01	2.85E-01	-0.580	0.546
KPNA2	0.136	1.174	0.190	0.500	2.77E-01	3.18E-01	-0.999	0.497
LOC728026, PTMA, PTMAP2	0.170	2.348	3.844	2.121	4.04E-01	4.56E-01	1.085	0.341
ROCK2	0.559	19.565	1.193	7.105	4.31E-01	4.78E-01	2.829	0.321
DDX21	0.681	0.978	5.665	2.441	4.66E-01	5.08E-01	1.288	0.294
UBR5	0.052	4.891	1.387	2.110	5.22E-01	5.59E-01	1.077	0.253
ZNRD1	0.272	0.945	1.281	0.833	6.30E-01	6.62E-01	-0.264	0.179
XRCC5	0.097	2.739	0.883	1.240	7.88E-01	8.15E-01	0.310	0.089
CAD	2.214	0.822	0.405	1.147	8.14E-01	8.27E-01	0.197	0.082
NOLC1	0.393	2.054	0.106	0.851	8.29E-01	8.29E-01	-0.233	0.081
POLR1C	1.000	1.000	1.000	1.000	N/A	N/A	0.000	N/A

Supplementary Table 6c: Quantification of the high-confidence interactors of the R279Q mutant POLR1C against WT POLR1C.

Protein Symbol	Quantification Ratio 1	Quantification Ratio 2	Quantification Ratio 3	Average Ratio	Two-tailed one-sample t-test p-value	BH-Adjusted p-value	Log2 (Average Ratio)	(-1)LOG10(BH-Adjusted p-value)
TUBA4A	0.015	0.020	0.012	0.016	6.23E-06	4.80E-04	-6.003	3.319
RPL13A	0.249	0.255	0.219	0.241	2.16E-04	8.32E-03	-2.055	2.080
ROCK2	0.174	0.255	0.109	0.179	2.61E-03	4.71E-02	-2.479	1.327
RPL28	0.290	0.347	0.219	0.285	2.70E-03	4.71E-02	-1.809	1.327
RPL21, RPL21P28	0.435	0.318	0.350	0.368	3.06E-03	4.71E-02	-1.443	1.327
GPN1	0.145	0.294	0.291	0.243	4.19E-03	5.38E-02	-2.038	1.269
ZNRD1	0.410	0.530	0.389	0.443	6.22E-03	6.84E-02	-1.175	1.165
POLR2K	0.498	0.159	0.350	0.335	2.11E-02	2.03E-01	-1.576	0.693
RPL35A	0.145	0.509	0.175	0.276	2.50E-02	2.14E-01	-1.855	0.669
POLR1E	0.484	0.449	0.125	0.353	2.98E-02	2.25E-01	-1.504	0.648
RPL27	0.581	0.636	0.350	0.522	3.21E-02	2.25E-01	-0.937	0.648
RPL13	0.092	0.662	0.168	0.307	6.05E-02	3.24E-01	-1.703	0.490
LOC101060521, POLR3E	0.783	0.833	0.604	0.740	6.45E-02	3.24E-01	-0.435	0.490
POLR2E	0.689	0.862	0.855	0.802	7.32E-02	3.24E-01	-0.318	0.490
RPL34	0.484	0.742	0.219	0.482	7.56E-02	3.24E-01	-1.054	0.490
TCOF1	0.332	0.799	0.466	0.533	7.80E-02	3.24E-01	-0.909	0.490
RPL4	0.272	0.773	0.228	0.424	8.10E-02	3.24E-01	-1.237	0.490
RPL18A	0.290	0.795	0.437	0.508	8.16E-02	3.24E-01	-0.978	0.490
POLR3H	0.523	0.801	0.787	0.704	8.21E-02	3.24E-01	-0.507	0.490
POLR2F	0.355	0.603	0.820	0.592	9.36E-02	3.24E-01	-0.755	0.490
POLR3F	0.587	0.906	0.630	0.708	1.00E-01	3.24E-01	-0.499	0.490
RPL27A, RPL27AP6	0.348	0.848	0.350	0.516	1.01E-01	3.24E-01	-0.956	0.490
RPL8	0.187	0.818	0.364	0.456	1.02E-01	3.24E-01	-1.132	0.490
RPS9	0.067	0.788	0.046	0.300	1.03E-01	3.24E-01	-1.736	0.490
POLR3B	0.797	0.760	0.418	0.658	1.05E-01	3.24E-01	-0.604	0.490
S100A11	0.871	0.587	0.291	0.583	1.30E-01	3.86E-01	-0.778	0.414
RPL26, RPL26P32	0.134	0.848	0.510	0.497	1.35E-01	3.86E-01	-1.007	0.414
RPL7	0.406	0.926	0.278	0.537	1.44E-01	3.94E-01	-0.898	0.405
LOC101929876, RPS26, RPS26P15, RPS26P25, RPS26P58	0.373	0.909	0.087	0.457	1.53E-01	3.94E-01	-1.131	0.405
POLR3C	0.669	0.961	0.554	0.728	1.53E-01	3.94E-01	-0.458	0.405
POLR3D	0.360	0.973	0.350	0.561	1.67E-01	4.15E-01	-0.834	0.382
POLR1A	0.907	0.870	0.527	0.768	1.96E-01	4.70E-01	-0.380	0.327
CD3EAP	0.330	0.571	1.004	0.635	2.06E-01	4.80E-01	-0.654	0.319
POLR1D	0.746	0.018	0.874	0.546	2.31E-01	5.23E-01	-0.873	0.281
POLR2L	0.809	1.000	0.546	0.785	2.44E-01	5.31E-01	-0.349	0.275

Protein Symbol	Quantification Ratio 1	Quantification Ratio 2	Quantification Ratio 3	Average Ratio	Two-tailed one-sample t-test p-value	BH-Adjusted p-value	Log2 (Average Ratio)	(-1)LOG10(BH-Adjusted p-value)
POLR3K	0.532	0.891	0.954	0.792	2.54E-01	5.31E-01	-0.336	0.275
POLR2H	0.594	1.026	0.787	0.802	2.55E-01	5.31E-01	-0.318	0.275
RPL3	0.653	1.030	0.119	0.601	2.70E-01	5.36E-01	-0.735	0.271
POLR1B	0.812	1.022	0.551	0.795	2.71E-01	5.36E-01	-0.331	0.271
RPL7A	0.279	1.152	0.336	0.589	2.82E-01	5.43E-01	-0.764	0.265
UBR5	1.393	15.273	3.716	6.794	3.10E-01	5.81E-01	2.764	0.236
TWISTNB	0.493	0.424	1.166	0.694	3.25E-01	5.83E-01	-0.526	0.234
RPL18	0.272	1.188	0.486	0.649	3.32E-01	5.83E-01	-0.625	0.234
PPP1CA, PPP1CC	0.653	17.818	3.497	7.323	3.56E-01	5.83E-01	2.872	0.234
POLR3A	0.883	1.043	0.476	0.801	3.59E-01	5.83E-01	-0.321	0.234
CRCP	0.644	0.933	1.020	0.866	3.59E-01	5.83E-01	-0.208	0.234
H3F3A, H3F3AP4, H3F3B	3.484	3.273	0.079	2.279	3.65E-01	5.83E-01	1.188	0.234
DDX21	2.613	3.564	0.233	2.136	3.70E-01	5.83E-01	1.095	0.234
PRKDC	2.177	11.964	0.874	5.005	3.71E-01	5.83E-01	2.323	0.234
RPS7	0.784	1.131	0.154	0.690	3.91E-01	6.03E-01	-0.536	0.220
RPS6	0.290	1.379	0.097	0.589	4.11E-01	6.14E-01	-0.764	0.212
H2AFJ, HIST1H2AG, HIST1H2AH, HIST1H2AI, HIST1H2AJ, HIST1H2AK, HIST1H2AL, HIST1H2AM	0.871	54.727	0.229	18.609	4.32E-01	6.14E-01	4.218	0.212
DHX9	0.871	46.455	0.051	15.792	4.36E-01	6.14E-01	3.981	0.212
ACIN1	0.435	36.909	0.049	12.464	4.47E-01	6.14E-01	3.640	0.212
PES1	1.161	7.000	0.583	2.915	4.49E-01	6.14E-01	1.543	0.212
RPL14	0.079	0.909	1.124	0.704	4.51E-01	6.14E-01	-0.506	0.212
RPLP0	0.109	1.352	0.538	0.666	4.57E-01	6.14E-01	-0.586	0.212
EPPK1	1.306	6.788	0.309	2.801	4.66E-01	6.14E-01	1.486	0.212
RPL15	0.327	1.273	0.669	0.756	4.71E-01	6.14E-01	-0.404	0.212
POLR2B	1.306	4.667	0.397	2.123	4.78E-01	6.14E-01	1.086	0.212
RPL7P32	6.096	0.926	0.306	2.443	5.14E-01	6.49E-01	1.288	0.188
NONO	0.290	9.164	0.060	3.171	5.44E-01	6.76E-01	1.665	0.170
RSL1D1	0.435	6.788	0.062	2.429	5.80E-01	7.09E-01	1.280	0.149
HIST1H2BC, HIST1H2BD, HIST1H2BE, HIST1H2BF, HIST1H2BG, HIST1H2BI, HIST1H2BK, HIST1H2BL	0.633	5.091	0.175	1.966	6.00E-01	7.12E-01	0.976	0.148
CCT7P2	1.451	0.636	1.399	1.162	6.01E-01	7.12E-01	0.217	0.148
RPL7L1	1.089	2.864	0.250	1.401	6.55E-01	7.64E-01	0.486	0.117
PRPF8	0.218	4.492	0.258	1.656	6.89E-01	7.92E-01	0.728	0.101
NOLC1	0.145	1.943	0.250	0.779	7.41E-01	8.39E-01	-0.360	0.076
XRCC5	1.219	2.000	0.300	1.173	7.58E-01	8.46E-01	0.230	0.073

Protein Symbol	Quantification Ratio 1	Quantification Ratio 2	Quantification Ratio 3	Average Ratio	Two-tailed one-sample t-test p-value	BH-Adjusted p-value	Log2 (Average Ratio)	(-1)LOG10(BH-Adjusted p-value)
KPNA2	0.871	2.545	0.269	1.228	7.69E-01	8.46E-01	0.297	0.073
RPS2, RPS2P5	0.290	1.980	0.199	0.823	7.89E-01	8.55E-01	-0.281	0.068
HIST1H1C	0.504	1.724	0.463	0.897	8.27E-01	8.68E-01	-0.157	0.061
POLR3G	0.653	0.821	1.366	0.947	8.28E-01	8.68E-01	-0.079	0.061
LRRIQ3	1.120	0.955	0.874	0.983	8.34E-01	8.68E-01	-0.025	0.061
MAF1	0.536	0.636	1.624	0.932	8.627E-01	8.857E-01	-0.102	0.053
SNRPB, SNRPN	1.306	1.591	0.291	1.063	8.880E-01	8.996E-01	0.088	0.046
HIST1H1E	0.504	1.886	0.463	0.951	9.259E-01	9.259E-01	-0.073	0.033
POLR1C	1.000	1.000	1.000	1.000	N/A	N/A	0.000	N/A

Supplementary Table 7: POLR1C ChIP-Sequencing data.

All peaks called by MACS and corresponding to known Pol III transcribed-genes are listed. Alu sequences and other repeats were not included. POLR1C variant (WT, N32I or N74S) is indicated in the first column. The gene full name was obtained in tRNAscan-SE or GENCODE Genes V19. Several peaks encompassed more than one neighboring tRNA genes, which are indicated as second, third and fourth genes. Legend: Chr: chromosome, Abs: absolute, FE: fold enrichment.

POLR1C variant	Chr	Start	End	Length	Abs summit	Pileup	-LOG10 (p-value)	FE	-LOG10 (q-value)	First Gene Full Name	Second Gene Full Name	Third Gene Full Name	Fourth Gene Full Name
WT	chr1	16861506	16862054	549	16861855	44	38.29	12.39	33.94	chr1.tRNA134-GluTTC			
WT	chr1	16872154	16872857	704	16872491	235	294.95	38.89	289.59	chr1.tRNA133-GlyCCC			
WT	chr1	16873931	16874466	536	16874204	108	94.23	15.66	89.68	chr1.tRNA132-PseudoCAC			
WT	chr1	17004434	17005098	665	17004785	196	217.87	28.31	212.86	chr1.tRNA131-GlyCCC			
WT	chr1	17053559	17054133	575	17053851	118	103.32	16.01	98.73	chr1.tRNA2-GlyCCC			
WT	chr1	17186431	17187034	604	17186736	103	112.14	23.61	107.52	chr1.tRNA3-PseudoCAC			
WT	chr1	17188165	17188884	720	17188453	184	146.67	14.32	141.93	chr1.tRNA4-GlyCCC			
WT	chr1	17199048	17199355	308	17199146	22	11.82	5.33	7.65	chr1.tRNA5-GluTTC			
WT	chr1	93981765	93982022	258	93981894	29	15.78	5.89	11.57	chr1.tRNA127-CysGCA			
WT	chr1	94312761	94313534	774	94313172	294	359.29	37.77	353.57	chr1.tRNA9-ArgTCT			
WT	chr1	145395199	145395931	733	145395570	348	548.81	77.41	542.00	chr1.tRNA119-LysCTT			
WT	chr1	145396546	145398291	1746	145397887	327	414.10	42.00	408.11	chr1.tRNA118-HisGTG	chr1.tRNA117-GlyTCC		
WT	chr1	145398728	145399613	886	145399248	375	460.66	39.53	454.41	chr1.tRNA116-GluCTC			
WT	chr1	147825517	147825919	403	147825709	35	28.79	10.54	24.50	chr1.tRNA23-PseudoCTG			
WT	chr1	148000623	148001290	668	148000886	50	36.72	10.01	32.38	chr1.tRNA25-AsnGTT			
WT	chr1	148247869	148248374	506	148248141	87	68.61	12.65	64.15	chr1.tRNA26-AsnGTT			
WT	chr1	148597947	148598653	707	148598325	194	203.90	24.97	198.94	chr1.tRNA108-AsnGTT			
WT	chr1	148759987	148760653	667	148760365	133	127.00	19.26	122.33	chr1.tRNA107-AsnGTT			
WT	chr1	149294210	149295011	802	149294690	288	258.20	18.68	253.01	chr1.tRNA99-ValCAC			
WT	chr1	149298249	149298892	644	149298589	220	242.53	28.30	237.41	chr1.tRNA98-ValCAC			
WT	chr1	149679784	149680583	800	149680272	404	537.44	49.32	530.70	chr1.tRNA91-PseudoCCC			
WT	chr1	149683699	149684467	769	149684176	313	390.66	40.21	384.78	chr1.tRNA90-ValCAC			
WT	chr1	153643388	153644155	768	153643752	350	380.99	29.09	375.15	chr1.tRNA32-MetCAT			

POLR1C variant	Chr	Start	End	Length	Abs summit	Pileup	-LOG10 (p-value)	FE	-LOG10 (q-value)	First Gene Full Name	Second Gene Full Name	Third Gene Full Name	Fourth Gene Full Name
WT	chr1	161369140	161369863	724	161369543	281	354.12	40.53	348.43	chr1.tRNA85-ValCAC			
WT	chr1	161391675	161392217	543	161391895	115	96.82	14.85	92.26	chr1.tRNA84-GluTTC			
WT	chr1	161397565	161398215	651	161397866	145	164.07	27.78	159.27	chr1.tRNA83-AsnGTT			
WT	chr1	161410372	161410794	423	161410620	85	50.86	8.30	46.46	chr1.tRNA81-AspGTC			
WT	chr1	161412810	161413513	704	161413140	242	264.20	28.06	258.98	chr1.tRNA35-GlyGCC			
WT	chr1	161416649	161417422	774	161417065	423	540.93	44.58	534.17	chr1.tRNA80-GluCTC			
WT	chr1	161417706	161418216	511	161418039	182	134.41	12.52	129.72	chr1.tRNA78-AspGTC			
WT	chr1	161420216	161420852	637	161420524	297	244.10	15.79	238.97	chr1.tRNA37-GlyGCC			
WT	chr1	161423924	161424828	905	161424445	506	515.99	25.70	509.42	chr1.tRNA77-GluCTC			
WT	chr1	161425140	161425592	453	161425410	179	158.49	17.37	153.71	chr1.tRNA75-AspGTC			
WT	chr1	161427604	161428352	749	161427923	252	248.80	22.56	243.66	chr1.tRNA39-GlyGCC			
WT	chr1	161431501	161432239	739	161431860	427	441.42	26.22	435.30	chr1.tRNA74-GluCTC			
WT	chr1	161432494	161433001	508	161432817	175	153.37	16.98	148.60	chr1.tRNA72-AspGTC			
WT	chr1	161434981	161435650	670	161435327	303	240.19	14.77	235.08	chr1.tRNA41-GlyGCC			
WT	chr1	161438873	161439620	748	161439237	536	521.87	23.22	515.24	chr1.tRNA71-GluCTC			
WT	chr1	161439875	161440359	485	161440186	160	147.82	18.59	143.07	chr1.tRNA69-AspGTC			
WT	chr1	161493332	161494005	674	161493682	255	327.85	41.92	322.32	chr1.tRNA68-GlyGCC			
WT	chr1	161499829	161501303	1475	161500966	229	202.53	17.81	197.57	chr1.tRNA67-LeuCAG	chr1.tRNA45-GlyTCC		
WT	chr1	161509738	161510400	663	161510085	200	223.97	28.89	218.94	chr1.tRNA47-AsnGTT			
WT	chr1	161582203	161582839	637	161582549	183	227.49	36.13	222.44	chr1.tRNA49-GluTTC			
WT	chr1	161591194	161591754	561	161591493	59	41.61	9.82	37.25	chr1.tRNA50-AsnGTT			
WT	chr1	167683609	167685146	1538	167684742	222	258.12	32.05	252.93	chr1.tRNA52-ProCGG	chr1.tRNA65-ProAGG		
WT	chr1	204475320	204476543	1224	204475694	355	461.74	45.59	455.49	chr1.tRNA54-LysTTT	chr1.tRNA62-LysTTT		
WT	chr1	222637997	222638671	675	222638375	163	144.67	17.24	139.93	chr1.tRNA56-ThrTGT			
WT	chr1	228745764	228746400	637	228746096	197	168.04	16.35	163.22	RNA5S1			
WT	chr1	228747855	228748662	808	228748303	173	128.85	12.64	124.18	RNA5S2			
WT	chr1	228750242	228750851	610	228750550	222	200.38	18.48	195.43	RNA5S3			
WT	chr1	228752326	228753118	793	228752767	165	147.98	17.62	143.24	RNA5S4			
WT	chr1	228754745	228755360	616	228755029	212	194.46	18.99	189.54	RNA5S5			
WT	chr1	228756945	228757595	651	228757225	187	155.94	15.58	151.17	RNA5S6			

POLR1C variant	Chr	Start	End	Length	Abs summit	Pileup	-LOG10 (p-value)	FE	-LOG10 (q-value)	First Gene Full Name	Second Gene Full Name	Third Gene Full Name	Fourth Gene Full Name
WT	chr1	228759118	228759783	666	228759483	186	167.54	18.04	162.73	RNA5S7			
WT	chr1	228761401	228762056	656	228761695	221	206.42	19.80	201.45	RNA5S8			
WT	chr1	228763631	228764268	638	228763980	136	121.33	16.96	116.68	RNA5S9			
WT	chr1	228765852	228766524	673	228766177	202	174.67	16.82	169.83	RNA5S10			
WT	chr1	228768125	228768751	627	228768429	207	212.76	24.02	207.77	RNA5S11			
WT	chr1	228770316	228771009	694	228770680	218	234.47	26.73	229.38	RNA5S12			
WT	chr1	228772606	228773226	621	228772924	192	141.11	12.48	136.39	RNA5S13			
WT	chr1	228774795	228775450	656	228775129	216	216.20	22.81	211.19	RNA5S14			
WT	chr1	228777096	228777646	551	228777390	132	109.56	14.79	104.96	RNA5S15			
WT	chr1	228779335	228779934	600	228779617	134	120.95	17.30	116.30	RNA5S16			
WT	chr1	228781547	228782199	653	228781830	211	173.70	15.40	168.86	RNA5S17			
WT	chr1	249167740	249168859	1120	249168111	240	344.23	54.71	338.60	chr1.tRNA58-LeuCAA	chr1.tRNA59-GluCTC		
WT	chr2	27273385	27274513	1129	27273698	366	462.32	42.37	456.06	chr2.tRNA2-TyrGTA	chr2.tRNA3-AlaAGC		
WT	chr2	43037348	43038110	763	43037748	303	374.09	38.93	368.28	chr2.tRNA5-IleTAT			
WT	chr2	47562279	47562752	474	47562483	54	36.33	9.01	32.00	BCYRN1			
WT	chr2	70475825	70476491	667	70476151	277	365.67	45.52	359.91	chr2.tRNA27-GlyCCC			
WT	chr2	131094411	131095111	701	131094743	202	312.03	62.83	306.59	chr2.tRNA20-GluTTC			
WT	chr2	157256969	157258063	1095	157257696	255	352.01	50.26	346.34	chr2.tRNA13-AlaCGC	chr2.tRNA19-GlyGCC		
WT	chr3	45730136	45730871	736	45730518	224	342.60	63.32	336.98	chr3.tRNA11-ArgACG			
WT	chr3	131947844	131948079	236	131948002	22	11.59	5.22	7.42	chr3.tRNA7-CysGCA			
WT	chr3	169489726	169490439	714	169490034	208	249.90	34.22	244.75	chr3.tRNA2-ValAAC			
WT	chr5	140090548	140091250	703	140090879	316	489.62	71.97	483.20	VTRNA1-1			
WT	chr5	140098240	140098912	673	140098552	304	360.51	35.22	354.78	VTRNA1-2			
WT	chr5	140105489	140106233	745	140105797	284	399.14	53.89	393.22	VTRNA1-3			
WT	chr5	180596531	180596838	308	180596729	24	17.41	7.74	13.18	chr5.tRNA5-ValAAC			
WT	chr5	180600551	180600908	358	180600685	31	17.08	6.09	12.86	chr5.tRNA6-ValCAC			
WT	chr5	180614406	180616277	1872	180615925	251	277.79	29.10	272.51	chr5.tRNA7-LeuAAG	chr5.tRNA15-ValAAC	chr5.tRNA14-ProTGG	
WT	chr5	180618332	180619042	711	180618732	262	360.25	50.04	354.53	chr5.tRNA13-ThrTGT			
WT	chr5	180633553	180635140	1588	180633906	304	360.51	35.22	354.78	chr5.tRNA8-AlaTGC	chr5.tRNA9-LysCTT		
WT	chr5	180644946	180645645	700	180645317	214	259.61	35.20	254.42	chr5.tRNA12-ValAAC			
WT	chr5	180648585	180649730	1146	180649420	179	252.55	48.52	247.39	chr5.tRNA11-LysCTT	chr5.tRNA10-ValCAC		
WT	chr6	26286451	26287112	662	26286790	283	376.12	46.50	370.30	chr6.tRNA2-MetCAT			
WT	chr6	26305498	26305912	415	26305653	26	15.09	6.13	10.89	chr6.tRNA175-SerGCT			
WT	chr6	26311222	26312286	1065	26311993	81	67.05	13.51	62.60	chr6.tRNA174-GlnTTG	chr6.tRNA173-GlnTTG		

POLR1C variant	Chr	Start	End	Length	Abs summit	Pileup	-LOG10 (p-value)	FE	-LOG10 (q-value)	First Gene Full Name	Second Gene Full Name	Third Gene Full Name	Fourth Gene Full Name
WT	chr6	26312610	26313768	1159	26313384	144	151.42	23.74	146.66	chr6.tRNA172-SerTGA	chr6.tRNA171-MetCAT		
WT	chr6	26322770	26323549	780	26323098	183	205.09	28.49	200.13	chr6.tRNA4-ArgTCG			
WT	chr6	26327519	26328778	1260	26327877	204	208.47	23.67	203.49	chr6.tRNA5-SerAGA	chr6.tRNA6-ArgACG		
WT	chr6	26330261	26330914	654	26330556	183	170.90	19.34	166.07	chr6.tRNA169-MetCAT			
WT	chr6	26331470	26331986	517	26331712	98	88.06	16.21	83.52	chr6.tRNA168-TrpCCA			
WT	chr6	26521291	26522004	714	26521515	208	311.71	58.81	306.27	chr6.tRNA7-LeuCAG			
WT	chr6	26532815	26533514	700	26533179	245	310.92	40.28	305.49	chr6.tRNA167-ThrAGT			
WT	chr6	26537457	26538622	1166	26537765	166	206.82	35.51	201.85	chr6.tRNA8-ArgACG	chr6.tRNA9-ValCAC		
WT	chr6	26553443	26554765	1323	26554382	201	204.19	23.32	199.23	chr6.tRNA10-AlaCGC	chr6.tRNA11-IleAAT		
WT	chr6	26555281	26555847	567	26555512	72	69.09	16.92	64.62	chr6.tRNA12-ProAGG			
WT	chr6	26556537	26557217	681	26556766	110	112.26	21.12	107.64	chr6.tRNA13-LysCTT			
WT	chr6	26568699	26569446	748	26569124	210	239.37	30.32	234.27	chr6.tRNA14-TyrGTA			
WT	chr6	26571817	26572513	697	26572134	184	199.79	26.59	194.85	chr6.tRNA166-AlaAGC			
WT	chr6	26577061	26577653	593	26577376	90	102.46	24.79	97.88	chr6.tRNA16-TyrGTA			
WT	chr6	26735355	26735885	531	26735594	92	87.10	17.59	82.57	chr6.tRNA164-MetCAT			
WT	chr6	26745019	26745518	500	26745274	61	57.69	15.72	53.27	chr6.tRNA163-IleAAT			
WT	chr6	26758416	26758838	423	26758579	58	50.57	13.55	46.18	chr6.tRNA162-MetCAT			
WT	chr6	26766133	26766778	646	26766429	70	49.45	10.20	45.06	chr6.tRNA27-MetCAT			
WT	chr6	26987910	26988404	495	26988145	79	59.40	11.50	54.97	chr6.tRNA29-IleTAT			
WT	chr6	27059270	27059931	662	27059523	120	126.63	23.02	121.96	chr6.tRNA30-ProCGG			
WT	chr6	27125699	27126195	497	27125921	53	43.42	11.98	39.06	chr6.tRNA33-HisGTG			
WT	chr6	27129860	27130360	501	27130101	83	69.22	13.75	64.76	chr6.tRNA34-ThrAGT			
WT	chr6	27144662	27145501	840	27145025	231	237.53	24.39	232.44	chr6.tRNA158-IleAAT			
WT	chr6	27181399	27181951	553	27181667	125	124.34	20.63	119.68	chr6.tRNA156-ArgACG			
WT	chr6	27182690	27183225	536	27182978	105	79.62	12.24	75.12	chr6.tRNA36-ArgACG			
WT	chr6	27197912	27198672	761	27198362	89	83.39	17.12	78.88	chr6.tRNA155-LeuTAA			
WT	chr6	27203122	27203372	251	27203297	18	10.40	5.35	6.24	chr6.tRNA37-ValAAC			

POLR1C variant	Chr	Start	End	Length	Abs summit	Pileup	-LOG10 (p-value)	FE	-LOG10 (q-value)	First Gene Full Name	Second Gene Full Name	Third Gene Full Name	Fourth Gene Full Name
WT	chr6	27205164	27205487	324	27205360	36	29.15	10.41	24.86	chr6.tRNA154-IleAAT			
WT	chr6	27247799	27248376	578	27248088	129	129.95	21.29	125.26	chr6.tRNA152-ValCAC			
WT	chr6	27261368	27261911	544	27261709	73	74.92	19.14	70.43	chr6.tRNA41-PseudoACT			
WT	chr6	27262973	27263510	538	27263250	63	45.96	10.48	41.59	chr6.tRNA42-GlnCTG			
WT	chr6	27265506	27266057	552	27265845	88	75.38	14.57	70.90	chr6.tRNA43-SerGCT			
WT	chr6	27271484	27271745	262	27271599	17	7.62	4.09	3.52	chr6.tRNA151-ThrCGT			
WT	chr6	27300671	27300976	306	27300775	34	19.79	6.66	15.55	chr6.tRNA150-MetCAT			
WT	chr6	27302533	27303058	526	27302833	63	64.14	18.01	59.69	chr6.tRNA149-LysTTT			
WT	chr6	27446340	27447845	1506	27447510	168	166.77	21.64	161.96	chr6.tRNA44-SerAGA	chr6.tRNA45-AspGTC		
WT	chr6	27463366	27463919	554	27463651	114	101.89	16.53	97.31	chr6.tRNA46-SerAGA			
WT	chr6	27470569	27471866	1298	27471577	105	124.07	27.99	119.41	chr6.tRNA47-SerAGA	chr6.tRNA48-AspGTC		
WT	chr6	27473367	27473875	509	27473660	63	61.07	16.56	56.64	chr6.tRNA148-SerTGA			
WT	chr6	27487026	27487682	657	27487337	200	237.06	32.91	231.97	chr6.tRNA49-GlnCTG			
WT	chr6	27515230	27515880	651	27515543	219	284.82	41.86	279.51	chr6.tRNA146-GlnCTG			
WT	chr6	27521050	27521456	407	27521270	38	23.58	7.42	19.31	chr6.tRNA145-SerAGA			
WT	chr6	27543678	27544246	569	27543878	38	33.66	12.07	29.34	chr6.tRNA53-LysTTT			
WT	chr6	27551003	27551546	544	27551302	108	88.28	13.96	83.75	chr6.tRNA144-AspGTC			
WT	chr6	27559381	27559865	485	27559625	79	73.25	16.33	68.78	chr6.tRNA143-LysTTT			
WT	chr6	27560168	27561040	873	27560655	173	194.79	28.49	189.87	chr6.tRNA142-MetCAT			
WT	chr6	27573313	27573702	390	27573527	32	20.81	7.49	16.56	chr6.tRNA140-LeuCAA			
WT	chr6	27585846	27586421	576	27586127	50	40.50	11.58	36.15	chr6.tRNA54-ThrCGT			
WT	chr6	27618390	27619073	684	27618768	139	154.90	26.64	150.13	chr6.tRNA139-ValAAC			
WT	chr6	27636139	27636752	614	27636422	86	72.90	14.25	68.43	chr6.tRNA57-IleAAT			
WT	chr6	27640043	27640640	598	27640214	28	18.66	7.39	14.42	chr6.tRNA137-SerCGA			
WT	chr6	27652091	27652738	648	27652481	137	164.88	31.33	160.07	chr6.tRNA135-ThrAGT			
WT	chr6	27655681	27656323	643	27656053	127	129.65	21.79	124.96	chr6.tRNA59-IleAAT			
WT	chr6	27694318	27694716	399	27694541	33	16.54	5.57	12.32	chr6.tRNA60-ThrAGT			

POLR1C variant	Chr	Start	End	Length	Abs summit	Pileup	-LOG10 (p-value)	FE	-LOG10 (q-value)	First Gene Full Name	Second Gene Full Name	Third Gene Full Name	Fourth Gene Full Name
WT	chr6	27720837	27721525	689	27721192	256	349.53	48.90	343.87	chr6.tRNA132-ValAAC			
WT	chr6	27745499	27745920	422	27745650	32	24.89	9.44	20.61	chr6.tRNA61-MetCAT			
WT	chr6	27758802	27759430	629	27759156	154	190.70	34.38	185.79	chr6.tRNA131-GlnCTG			
WT	chr6	27763335	27764030	696	27763668	233	240.41	24.60	235.30	chr6.tRNA130-GlnTTG			
WT	chr6	27869979	27871084	1106	27870745	251	304.56	36.22	299.15	chr6.tRNA129-MetCAT	chr6.tRNA128-GlyGCC		
WT	chr6	28180560	28181239	680	28180828	230	322.84	51.23	317.35	chr6.tRNA62-SerGCT			
WT	chr6	28442081	28442688	608	28442343	189	219.64	31.11	214.62	chr6.tRNA127-ThrTGT			
WT	chr6	28456424	28457114	691	28456820	104	132.40	32.50	127.71	chr6.tRNA125-ThrCGT			
WT	chr6	28510540	28511267	728	28510930	195	187.10	20.61	182.21	chr6.tRNA124-ArgTCG			
WT	chr6	28556875	28557604	730	28557207	99	77.59	12.80	73.09	chr6.tRNA64-GlnTTG			
WT	chr6	28564706	28565526	821	28565170	151	161.68	24.89	156.88	chr6.tRNA123-SerGCT			
WT	chr6	28574688	28575282	595	28574987	106	116.62	24.29	111.99	chr6.tRNA65-AlaAGC			
WT	chr6	28610975	28611539	565	28611237	128	120.27	18.54	115.63	chr6.tRNA66-AlaTGC			
WT	chr6	28615688	28616372	685	28615981	201	204.19	23.32	199.23	chr6.tRNA121-ThrCGT			
WT	chr6	28625737	28626363	627	28626044	281	324.07	32.56	318.57	chr6.tRNA120-AlaAGC			
WT	chr6	28641306	28641950	645	28641623	191	253.40	42.58	248.23	chr6.tRNA119-AlaCGC			
WT	chr6	28678184	28678653	470	28678392	41	30.24	9.54	25.94	chr6.tRNA67-AlaAGC			
WT	chr6	28687332	28687775	444	28687516	30	19.91	7.53	15.67	chr6.tRNA68-AlaAGC			
WT	chr6	28693717	28694060	344	28693861	21	12.01	5.61	7.84	chr6.tRNA69-ThrAGT			
WT	chr6	28696811	28697433	623	28697106	167	214.48	38.14	209.48	chr6.tRNA70-AlaCGC			
WT	chr6	28703038	28703509	472	28703192	37	14.35	4.39	10.15	chr6.tRNA115-ValAAC			
WT	chr6	28710502	28711013	512	28710727	62	55.23	14.30	50.82	chr6.tRNA114-ArgCCG			
WT	chr6	28715306	28715948	643	28715568	111	117.79	22.86	113.16	chr6.tRNA71-LysTTT			
WT	chr6	28725914	28726435	522	28726164	86	62.76	11.14	58.33	chr6.tRNA113-AlaTGC			
WT	chr6	28757282	28757864	583	28757559	137	164.88	31.33	160.07	chr6.tRNA110-AlaTGC			
WT	chr6	28758268	28758781	514	28758519	53	44.08	12.26	39.71	chr6.tRNA109-PheGAA			
WT	chr6	28763485	28764092	608	28763768	149	199.92	40.87	194.98	chr6.tRNA108-AlaAGC			

POLR1C variant	Chr	Start	End	Length	Abs summit	Pileup	-LOG10 (p-value)	FE	-LOG10 (q-value)	First Gene Full Name	Second Gene Full Name	Third Gene Full Name	Fourth Gene Full Name
WT	chr6	28770231	28770881	651	28770597	73	78.49	20.82	73.99	chr6.tRNA107-AlaTGC			
WT	chr6	28775348	28775960	613	28775666	105	128.00	29.83	123.33	chr6.tRNA106-PheGAA			
WT	chr6	28779581	28780094	514	28779828	41	35.13	11.82	30.80	chr6.tRNA105-AlaAGC			
WT	chr6	28784800	28785349	550	28785051	97	66.49	10.30	62.04	chr6.tRNA104-AlaTGC			
WT	chr6	28805934	28806653	720	28806266	233	309.01	44.52	303.58	chr6.tRNA102-AlaAGC			
WT	chr6	28831209	28831828	620	28831473	206	179.74	17.16	174.88	chr6.tRNA101-AlaAGC			
WT	chr6	28848875	28849477	603	28849152	170	217.39	37.93	212.38	chr6.tRNA73-ArgCCG			
WT	chr6	28863583	28864386	804	28864058	301	430.35	57.11	424.29	chr6.tRNA100-LeuCAA			
WT	chr6	28908551	28909758	1208	28908864	233	303.82	42.68	298.42	chr6.tRNA74-LeuCAA	chr6.tRNA99-GlnCTG		
WT	chr6	28911168	28912748	1581	28912393	196	245.93	37.48	240.79	chr6.tRNA98-LeuAAG	chr6.tRNA75-MetCAT		
WT	chr6	28918514	28919184	671	28918865	225	250.11	28.94	244.96	chr6.tRNA76-LysTTT			
WT	chr6	28920751	28921383	633	28921103	194	245.83	38.28	240.69	chr6.tRNA97-MetCAT			
WT	chr6	28949183	28950377	1195	28950011	263	383.86	58.55	378.01	chr6.tRNA96-PheGAA	chr6.tRNA77-GluCTC		
WT	chr6	52860123	52860955	833	52860469	252	342.42	48.14	336.80	RN7SK			
WT	chr6	126101073	126101812	740	126101441	364	458.96	42.14	452.72	chr6.tRNA87-GluCTC			
WT	chr6	144537379	144538050	672	144537723	241	323.00	46.04	317.50	chr6.tRNA83-LeuTAA			
WT	chr7	128423228	128423874	647	128423545	123	131.02	23.59	126.33	chr7.tRNA2-ProAGG			
WT	chr7	139025159	139025780	622	139025472	198	281.13	50.72	275.84	chr7.tRNA3-ArgCCT			
WT	chr7	148638252	148638926	675	148638600	269	325.34	36.33	319.83	RNY5			
WT	chr7	148660115	148660774	660	148660413	245	269.51	28.60	264.27	RNY4			
WT	chr7	148680571	148681203	633	148680903	198	233.87	32.58	228.79	RNY3			
WT	chr7	148683899	148684667	769	148684280	271	398.93	60.33	393.01	RNY1			
WT	chr7	149007125	149007497	373	149007341	38	20.85	6.39	16.60	chr7.tRNA5-CysGCA			
WT	chr7	149028116	149028446	331	149028214	22	11.59	5.22	7.42	chr7.tRNA6-CysGCA			
WT	chr7	149052395	149053114	720	149052794	49	39.33	11.35	34.98	chr7.tRNA25-CysGCA			
WT	chr7	149343835	149344328	494	149344042	52	45.33	13.12	40.96	chr7.tRNA17-CysGCA			
WT	chr7	149387907	149388584	678	149388355	69	64.29	15.89	59.85	chr7.tRNA16-CysGCA			
WT	chr7	149404552	149405025	474	149404785	84	70.45	13.92	65.98	chr7.tRNA15-CysGCA			
WT	chr8	67024989	67026743	1755	67025642	268	350.10	44.05	344.44	chr8.tRNA4-TyrGTA	chr8.tRNA5-TyrGTA	chr8.tRNA6-AlaAGC	

POLR1C variant	Chr	Start	End	Length	Abs summit	Pileup	-LOG10 (p-value)	FE	-LOG10 (q-value)	First Gene Full Name	Second Gene Full Name	Third Gene Full Name	Fourth Gene Full Name
WT	chr8	96281604	96282258	655	96281920	239	259.70	27.71	254.50	chr8.tRNA11-SerAGA			
WT	chr8	124169122	124169855	734	124169498	216	308.49	52.69	303.07	chr8.tRNA10-MetCAT			
WT	chr9	14433838	14434129	292	14433976	21	14.31	6.81	10.11	chr9.tRNA7-HisGTG			
WT	chr9	35657431	35658262	832	35657867	132	144.35	25.30	139.62	RMRP			
WT	chr9	112960696	112961030	335	112960824	21	10.75	4.99	6.59	chr9.tRNA2-ArgTCG			
WT	chr9	131102056	131102655	600	131102400	87	74.14	14.41	69.66	chr9.tRNA4-ArgTCT			
WT	chr9	137029347	137029929	583	137029637	149	149.06	21.56	144.31	RNU6ATAC			
WT	chr10	5895432	5895939	508	5895741	52	42.88	12.03	38.52	chr10.tRNA6-ValTAC			
WT	chr10	22518090	22518794	705	22518499	251	340.64	47.95	335.04	chr10.tRNA4-AsnGTT			
WT	chr10	23286013	23286378	366	23286188	23	15.75	7.10	11.54	RNA5SP304			
WT	chr10	69523954	69524671	718	69524314	257	351.31	49.09	345.65	chr10.tRNA2-SerTGA			
WT	chr11	59317726	59319540	1815	59318119	149	170.24	28.54	165.42	chr11.tRNA17-ValTAC	chr11.tRNA16-ValTAC	chr11.tRNA3-ArgTCT	chr11.tRNA4-LeuTAA
WT	chr11	59323674	59324244	571	59323928	112	132.95	28.80	128.26	chr11.tRNA5-LysTTT			
WT	chr11	59324671	59325260	590	59325006	100	107.70	22.93	103.10	chr11.tRNA15-PheGAA			
WT	chr11	59327486	59328196	711	59327845	265	344.94	43.56	339.31	chr11.tRNA14-LysTTT			
WT	chr11	59333467	59334366	900	59333860	175	167.97	20.32	163.16	chr11.tRNA13-PheGAA			
WT	chr11	66115311	66116033	723	66115629	306	416.66	50.27	410.67	chr11.tRNA8-SerGCT			
WT	chr11	75946168	75947295	1128	75946586	416	529.07	43.84	522.38	chr11.tRNA9-ProAGG	chr11.tRNA12-ProTGG		
WT	chr12	56583852	56584475	624	56584154	133	171.05	35.94	166.22	chr12.tRNA2-SerCGA			
WT	chr12	96429469	96430140	672	96429852	265	423.91	74.86	417.88	chr12.tRNA4-AspGTC			
WT	chr12	98896948	98898372	1425	98897339	322	424.06	46.42	418.03	chr12.tRNA5-AspGTC	chr12.tRNA6-TrpCCA		
WT	chr12	125405921	125406713	793	125406322	214	245.59	30.90	240.46	chr12.tRNA13-AlaTGC			
WT	chr12	125411506	125412796	1291	125412428	281	411.38	59.95	405.40	chr12.tRNA12-AspGTC	chr12.tRNA11-PheGAA		
WT	chr12	125423753	125425036	1284	125424382	249	301.30	35.93	295.92	chr12.tRNA10-AspGTC	chr12.tRNA8-AlaTGC		
WT	chr13	31247671	31248413	743	31248154	144	176.22	32.92	171.37	chr13.tRNA7-AsnGTT			
WT	chr13	41634639	41635168	530	41634896	76	87.28	23.83	82.76	chr13.tRNA5-GluTTC			
WT	chr13	45491709	45492419	711	45492063	200	271.72	45.63	266.47	chr13.tRNA3-GluTTC			
WT	chr13	95201494	95202293	800	95201940	253	344.19	48.33	338.57	chr13.tRNA1-PheGAA			
WT	chr14	20811184	20811751	568	20811545	45	36.81	11.38	32.48	RPPH1			

POLR1C variant	Chr	Start	End	Length	Abs summit	Pileup	-LOG10 (p-value)	FE	-LOG10 (q-value)	First Gene Full Name	Second Gene Full Name	Third Gene Full Name	Fourth Gene Full Name
WT	chr14	21077153	21078748	1596	21078359	250	319.36	41.10	313.89	chr14.tRNA23-ProAGG	chr14.tRNA1-LeuAAG		
WT	chr14	21081229	21082301	1073	21081989	225	250.11	28.94	244.96	chr14.tRNA22-ProAGG	chr14.tRNA21-ThrTGT		
WT	chr14	21093219	21094029	811	21093602	218	251.84	31.47	246.68	chr14.tRNA2-LeuTAG			
WT	chr14	21099023	21099585	563	21099333	122	112.31	17.68	107.69	chr14.tRNA20-ThrTGT			
WT	chr14	21100845	21101619	775	21101265	126	135.43	24.16	130.73	chr14.tRNA3-ProTGG			
WT	chr14	21149524	21150284	761	21149903	241	322.22	45.76	316.72	chr14.tRNA4-ThrTGT			
WT	chr14	21151140	21152519	1380	21151487	180	240.19	42.54	235.08	chr14.tRNA5-TyrGTA	chr14.tRNA6-ProTGG		
WT	chr14	23398560	23399332	773	23398927	349	358.48	25.42	352.76	chr14.tRNA7-ArgACG			
WT	chr14	32671165	32671497	333	32671346	27	20.66	8.67	16.41	RNU6-7			
WT	chr14	32672026	32672790	765	32672483	125	161.17	35.46	156.38	RNU6-8			
WT	chr14	50053035	50053807	773	50053310	119	125.18	22.83	120.51	RN7SL1			
WT	chr14	50329065	50329832	768	50329524	93	97.48	21.34	92.92	RN7SL2			
WT	chr14	58706340	58706998	659	58706651	253	307.81	36.50	302.39	chr14.tRNA13-LysCTT			
WT	chr14	73429417	73430167	751	73429739	127	148.95	29.06	144.20	chr14.tRNA8-CysGCA			
WT	chr14	102783049	102783798	750	102783469	284	342.99	36.49	337.37	chr14.tRNA10-IleAAT			
WT	chr15	26327228	26327564	337	26327393	22	15.32	7.12	11.12	chr15.tRNA11-GluTTC			
WT	chr15	40885717	40886426	710	40886079	268	317.23	34.44	311.76	chr15.tRNA10-SerGCT			
WT	chr15	45490394	45491178	785	45490848	222	289.97	42.43	284.64	chr15.tRNA9-HisGTG			
WT	chr15	45492315	45493768	1454	45492634	190	254.11	43.36	248.94	chr15.tRNA8-HisGTG	chr15.tRNA1-HisGTG		
WT	chr15	66161113	66161802	690	66161430	258	276.97	27.23	271.70	chr15.tRNA7-GlnCTG			
WT	chr15	68131970	68132720	751	68132314	124	122.95	20.47	118.30	RNU6-1			
WT	chr15	79152625	79153271	647	79152960	250	393.82	70.63	387.92	chr15.tRNA2-LysCTT			
WT	chr15	80036751	80037249	499	80037027	81	80.51	18.62	76.00	chr15.tRNA3-CysGCA			
WT	chr15	89877932	89878873	942	89878335	451	687.66	74.01	679.29	chr15.tRNA4-ArgTCG			
WT	chr16	686363	687088	726	686757	256	349.53	48.90	343.87	chr16.tRNA34-GlyCCC			
WT	chr16	3200380	3201113	734	3200701	143	140.70	20.69	135.98	chr16.tRNA1-ArgCCG			
WT	chr16	3202585	3203321	737	3202931	147	190.71	37.72	185.80	chr16.tRNA2-ArgCCT			
WT	chr16	3207004	3207765	762	3207463	110	103.84	18.18	99.25	chr16.tRNA32-LysCTT			
WT	chr16	3208739	3209207	469	3208964	74	70.95	17.03	66.48	chr16.tRNA3-ProTGG			

POLR1C variant	Chr	Start	End	Length	Abs summit	Pileup	-LOG10 (p-value)	FE	-LOG10 (q-value)	First Gene Full Name	Second Gene Full Name	Third Gene Full Name	Fourth Gene Full Name
WT	chr16	3225580	3225907	328	3225746	22	15.32	7.12	11.12	chr16.tRNA7-LysCTT			
WT	chr16	3232441	3232986	546	3232713	77	62.00	12.77	57.57	chr16.tRNA29-ProAGG			
WT	chr16	3233962	3234422	461	3234220	34	28.76	10.83	24.46	chr16.tRNA28-ProTGG			
WT	chr16	3239398	3239956	559	3239680	90	109.38	28.16	104.77	chr16.tRNA9-ProAGG			
WT	chr16	3241531	3242113	583	3241946	25	18.48	8.05	14.24	chr16.tRNA11-ProAGG			
WT	chr16	3245996	3246401	406	3246206	20	13.31	6.50	9.12	chr16.tRNA13-PseudoCTT			
WT	chr16	14379491	14380264	774	14379799	211	283.73	45.07	278.43	chr16.tRNA15-ThrCGT			
WT	chr16	22206611	22207423	813	22207095	258	409.82	72.88	403.85	chr16.tRNA27-LeuTAG			
WT	chr16	22308132	22308857	726	22308494	249	360.96	56.76	355.23	chr16.tRNA16-LeuAAG			
WT	chr16	57333562	57334805	1244	57334442	269	351.82	44.21	346.15	chr16.tRNA17-LeuCAG	chr16.tRNA26-LeuCAG		
WT	chr16	70811933	70812366	434	70812138	30	23.46	9.28	19.19	chr16.tRNA25-GlyGCC			
WT	chr16	70812651	70813306	656	70812988	185	147.87	14.40	143.12	chr16.tRNA24-GlyGCC			
WT	chr16	70823184	70823638	455	70823384	27	20.66	8.67	16.41	chr16.tRNA19-GlyGCC			
WT	chr16	73512028	73512536	509	73512294	37	32.42	11.76	28.11	chr16.tRNA23-LysTTT			
WT	chr16	87417358	87417957	600	87417699	157	217.04	44.46	212.03	chr16.tRNA22-MetCAT			
WT	chr17	8022088	8024638	2551	8022482	236	366.08	66.69	360.32	chr17.tRNA2-LysTTT	chr17.tRNA3-GlnCTG	chr17.tRNA42-LeuTAG	chr17.tRNA4-ArgTCT
WT	chr17	8028803	8029427	625	8029085	146	129.55	16.97	124.87	chr17.tRNA5-GlyGCC			
WT	chr17	8041931	8043093	1163	8042244	114	101.89	16.53	97.31	chr17.tRNA41-SerCGA	chr17.tRNA40-ThrAGT		
WT	chr17	8089428	8091345	1918	8090241	192	250.69	41.03	245.54	chr17.tRNA6-TrpCCA	chr17.tRNA7-SerGCT	chr17.tRNA8-ThrAGT	chr17.tRNA9-IleAAT
WT	chr17	8123957	8126524	2568	8125567	180	269.45	56.02	264.21	chr17.tRNA39-TrpCCA	chr17.tRNA10-GlyTCC	chr17.tRNA38-AspGTC	chr17.tRNA37-ProCGG
WT	chr17	8129353	8130542	1190	8129578	84	81.71	18.07	77.20	chr17.tRNA36-ThrAGT	chr17.tRNA35-SerAGA	chr17.tRNA34-IleAAT	
WT	chr17	19411184	19411866	683	19411509	269	318.83	34.57	313.35	chr17.tRNA12-TrpCCA			
WT	chr17	29876803	29877592	790	29877170	169	238.75	47.84	233.65	chr17.tRNA14-ThrCGT			
WT	chr17	36907763	36908391	629	36908059	203	274.43	45.25	269.17	chr17.tRNA31-AsnGTT			
WT	chr17	37023674	37024231	558	37023924	205	209.90	23.79	204.91	chr17.tRNA15-CysGCA			
WT	chr17	37025020	37026035	1016	37025624	160	175.07	26.36	170.22	chr17.tRNA28-CysGCA			
WT	chr17	37309639	37311143	1505	37310744	262	381.98	58.33	376.14	chr17.tRNA28-CysGCA			

POLR1C variant	Chr	Start	End	Length	Abs summit	Pileup	-LOG10 (p-value)	FE	-LOG10 (q-value)	First Gene Full Name	Second Gene Full Name	Third Gene Full Name	Fourth Gene Full Name
WT	chr17	47269097	47270310	1214	47269931	272	357.00	44.70	351.30	chr17.tRNA16-GlnTTG			
WT	chr17	66015671	66016337	667	66016023	326	396.06	37.76	390.15	chr17.tRNA23-ArgCCG			
WT	chr17	73029695	73031701	2007	73031251	270	374.64	51.56	368.83	chr17.tRNA18-ArgCCT	chr17.tRNA21-ArgCCT	chr17.tRNA19-ArgTCG	
WT	chr17	80452120	80452981	862	80452628	294	359.29	37.77	353.57	chr17.tRNA20-MetCAT			
WT	chr19	893205	893878	674	893474	167	185.63	27.51	180.75	RNU6-9			
WT	chr19	1383020	1383926	907	1383550	180	248.49	46.13	243.34	chr19.tRNA14-PheGAA	chr19.tRNA1-AsnGTT		
WT	chr19	4723799	4725070	1272	4724119	236	255.21	27.36	250.04	chr19.tRNA2-GlyTCC	chr19.tRNA13-ValCAC		
WT	chr19	33667610	33668515	906	33667996	170	250.46	52.93	245.31	chr19.tRNA4-ThrAGT			
WT	chr19	39902728	39903127	400	39902912	57	59.11	17.95	54.69	chr19.tRNA10-IleTAT			
WT	chr19	45981626	45982273	648	45981942	96	119.14	30.02	114.50	chr19.tRNA8-SeCTCA			
WT	chr19	48410853	48411137	285	48411006	33	13.62	4.55	9.43	SNAR-A12			
WT	chr19	48421535	48421957	423	48421700	43	36.53	11.86	32.19	SNAR-A1			
WT	chr19	48426922	48427238	317	48427061	39	21.12	6.33	16.87	SNAR-A3			
WT	chr19	48437279	48437635	357	48437479	44	34.61	10.67	30.29	SNAR-A2			
WT	chr19	48442628	48442989	362	48442866	66	19.35	3.72	15.11	SNAR-C4			
WT	chr19	48448082	48448362	281	48448244	28	11.60	4.40	7.43	SNAR-A13			
WT	chr19	48453242	48453760	519	48453474	40	7.39	2.51	3.30	SNAR-C3			
WT	chr19	48458789	48459126	338	48458947	50	20.63	4.92	16.38	SNAR-C2			
WT	chr19	49540152	49540482	331	49540280	23	15.08	6.75	10.88	SNAR-G1			
WT	chr19	50595649	50596009	361	50595834	44	29.44	8.51	25.15	SNAR-A4			
WT	chr19	50600908	50601404	497	50601126	49	45.21	14.07	40.84	SNAR-A5			
WT	chr19	50604013	50604481	469	50604197	46	35.85	10.67	31.52	SNAR-A6			
WT	chr19	50607075	50607530	456	50607294	40	33.91	11.54	29.59	SNAR-A7			
WT	chr19	50610128	50610605	478	50610373	44	37.62	12.07	33.28	SNAR-A8			
WT	chr19	50615480	50615926	447	50615720	54	45.90	12.75	41.53	SNAR-A9			
WT	chr19	50620894	50621274	381	50621080	39	29.57	9.76	25.28	SNAR-A10			
WT	chr19	50626145	50626630	486	50626422	49	42.43	12.74	38.07	SNAR-A11			
WT	chr19	50631492	50631945	454	50631753	50	43.67	13.00	39.31	SNAR-A14			
WT	chr19	50636946	50637253	308	50637108	34	15.39	5.03	11.19	SNAR-B2			
WT	chr21	18826662	18827405	744	18827113	167	185.63	27.51	180.75	chr21.tRNA2-GlyGCC			
WT	chrX	18692734	18693443	710	18693053	204	315.96	63.45	310.50	chrX.tRNA4-ValTAC			
N32I	chr1	16861718	16861954	237	16861805	16	8.03	4.61	3.71	chr1.tRNA134-GluTTC			
N32I	chr1	16872179	16872730	552	16872489	89	85.70	18.15	80.28	chr1.tRNA133-GlyCCC			
N32I	chr1	16874013	16874272	260	16874203	27	14.30	5.70	9.87	chr1.tRNA132-PseudoCAC			
N32I	chr1	17004590	17005027	438	17004786	57	51.98	14.54	47.00	chr1.tRNA131-GlyCCC			
N32I	chr1	17053645	17054076	432	17053850	50	33.54	8.92	28.83	chr1.tRNA2-GlyCCC			

POLR1C variant	Chr	Start	End	Length	Abs summit	Pileup	-LOG10 (p-value)	FE	-LOG10 (q-value)	First Gene Full Name	Second Gene Full Name	Third Gene Full Name	Fourth Gene Full Name
N32I	chr1	17186535	17186951	417	17186749	31	22.48	8.74	17.93	chr1.tRNA3-PseudoCAC			
N32I	chr1	17188232	17188721	490	17188480	70	67.51	16.98	62.29	chr1.tRNA4-GlyCCC			
N32I	chr1	94312947	94313400	454	94313176	71	70.40	18.01	65.14	chr1.tRNA9-ArgTCT			
N32I	chr1	145395306	145395828	523	145395564	117	135.16	27.77	129.24	chr1.tRNA119-LysCTT			
N32I	chr1	145396659	145397216	558	145396942	66	60.25	15.29	55.14	chr1.tRNA118-HisGTG			
N32I	chr1	145397674	145398191	518	145397913	126	131.76	22.96	125.87	chr1.tRNA117-GlyTCC			
N32I	chr1	145398952	145399506	555	145399262	117	97.96	14.78	92.43	chr1.tRNA116-GluCTC			
N32I	chr1	148000703	148001279	577	148000882	23	13.59	6.04	9.16	chr1.tRNA25-AsnGTT			
N32I	chr1	148247995	148248282	288	148248130	26	13.45	5.41	9.03	chr1.tRNA26-AsnGTT			
N32I	chr1	148598080	148598547	468	148598325	49	25.20	6.28	20.61	chr1.tRNA108-AsnGTT			
N32I	chr1	148760097	148760592	496	148760361	41	27.56	8.59	22.93	chr1.tRNA107-AsnGTT			
N32I	chr1	149294476	149294891	416	149294682	62	36.81	7.93	32.05	chr1.tRNA99-ValCAC			
N32I	chr1	149298359	149298830	472	149298604	65	39.95	8.36	35.14	chr1.tRNA98-ValCAC			
N32I	chr1	149680005	149680536	532	149680267	109	88.25	13.87	82.80	chr1.tRNA91-PseudoCCC			
N32I	chr1	149683900	149684368	469	149684173	83	54.09	9.36	49.09	chr1.tRNA90-ValCAC			
N32I	chr1	153643492	153644020	529	153643784	98	110.53	25.08	104.87	chr1.tRNA32-MetCAT			
N32I	chr1	161369304	161369784	481	161369560	83	78.76	17.42	73.40	chr1.tRNA85-ValCAC			
N32I	chr1	161391730	161392059	330	161391907	35	20.48	6.84	15.95	chr1.tRNA84-GluTTC			
N32I	chr1	161397604	161398099	496	161397899	47	44.30	14.56	39.43	chr1.tRNA83-AsnGTT			
N32I	chr1	161410517	161410761	245	161410625	33	17.06	5.81	12.59	chr1.tRNA81-AspGTC			
N32I	chr1	161412884	161413419	536	161413138	102	65.29	9.43	60.11	chr1.tRNA35-GlyGCC			
N32I	chr1	161416756	161417352	597	161417035	175	156.24	17.68	150.06	chr1.tRNA80-GluCTC			
N32I	chr1	161417804	161418187	384	161418028	75	46.74	8.73	41.84	chr1.tRNA78-AspGTC			
N32I	chr1	161420256	161420798	543	161420516	120	96.20	13.73	90.68	chr1.tRNA37-GlyGCC			
N32I	chr1	161424137	161424760	624	161424438	219	175.26	14.68	168.87	chr1.tRNA77-GluCTC			
N32I	chr1	161425172	161425545	374	161425382	65	36.55	7.35	31.80	chr1.tRNA75-AspGTC			
N32I	chr1	161427676	161428218	543	161427948	104	59.88	8.15	54.78	chr1.tRNA39-GlyGCC			

POLR1C variant	Chr	Start	End	Length	Abs summit	Pileup	-LOG10 (p-value)	FE	-LOG10 (q-value)	First Gene Full Name	Second Gene Full Name	Third Gene Full Name	Fourth Gene Full Name
N32I	chr1	161431518	161432157	640	161431853	154	106.63	11.13	101.01	chr1.tRNA74-GluCTC			
N32I	chr1	161432664	161432948	285	161432823	70	40.15	7.64	35.34	chr1.tRNA72-AspGTC			
N32I	chr1	161435044	161435547	504	161435311	121	80.68	10.21	75.30	chr1.tRNA41-GlyGCC			
N32I	chr1	161438874	161439541	668	161439236	209	184.09	17.51	177.60	chr1.tRNA71-GluCTC			
N32I	chr1	161439954	161440301	348	161440141	64	35.63	7.27	30.89	chr1.tRNA69-AspGTC			
N32I	chr1	161493468	161493968	501	161493662	92	91.01	19.20	85.54	chr1.tRNA68-GlyGCC			
N32I	chr1	161499906	161501216	1311	161500140	93	111.43	27.80	105.75	chr1.tRNA67-LeuCAG	chr1.tRNA45-GlyTCC		
N32I	chr1	161509826	161510324	499	161510076	67	63.48	16.29	58.33	chr1.tRNA47-AsnGTT			
N32I	chr1	161582262	161582780	519	161582521	59	54.57	15.08	49.56	chr1.tRNA49-GluTTC			
N32I	chr1	167683735	167684277	543	167684002	50	48.74	15.66	43.80	chr1.tRNA52-ProCGG			
N32I	chr1	167684527	167684963	437	167684763	63	59.85	16.17	54.75	chr1.tRNA65-ProAGG			
N32I	chr1	204475436	204476532	1097	204476200	120	125.73	22.85	119.91	chr1.tRNA54-LysTTT	chr1.tRNA62-LysTTT		
N32I	chr1	222638146	222638610	465	222638382	65	52.57	12.54	47.58	chr1.tRNA56-ThrTGT			
N32I	chr1	228745832	228746238	407	228746075	68	31.08	5.81	26.40	RNA5S1			
N32I	chr1	228748079	228748501	423	228748275	82	45.07	7.41	40.19	RNA5S2			
N32I	chr1	228750309	228750780	472	228750518	73	25.26	4.40	20.67	RNA5S3			
N32I	chr1	228752602	228752902	301	228752735	63	25.16	4.98	20.57	RNA5S4			
N32I	chr1	228754835	228755289	455	228755062	69	40.05	7.77	35.24	RNA5S5			
N32I	chr1	228756933	228757378	446	228757213	68	26.93	4.98	22.31	RNA5S6			
N32I	chr1	228759266	228759643	378	228759455	69	29.67	5.42	25.01	RNA5S7			
N32I	chr1	228761515	228761865	351	228761662	76	30.90	5.18	26.23	RNA5S8			
N32I	chr1	228763768	228764071	304	228763885	60	26.97	5.62	22.35	RNA5S9			
N32I	chr1	228766006	228766424	419	228766205	65	26.64	5.14	22.03	RNA5S10			
N32I	chr1	228768225	228768653	429	228768408	65	36.55	7.35	31.80	RNA5S11			
N32I	chr1	228770463	228770904	442	228770633	63	27.15	5.39	22.53	RNA5S12			
N32I	chr1	228772688	228773114	427	228772903	66	38.98	7.91	34.19	RNA5S13			
N32I	chr1	228774954	228775303	350	228775136	62	27.98	5.68	23.34	RNA5S14			
N32I	chr1	228777150	228777536	387	228777333	46	18.93	4.87	14.43	RNA5S15			
N32I	chr1	228779486	228779789	304	228779633	46	16.65	4.30	12.19	RNA5S16			
N32I	chr1	228781622	228782060	439	228781852	73	33.12	5.82	28.41	RNA5S17			
N32I	chr1	249167837	249168759	923	249168149	62	65.51	19.39	60.33	chr1.tRNA58-LeuCAA	chr1.tRNA59-GluCTC		
N32I	chr2	27273417	27274328	912	27273674	135	174.30	36.49	167.91	chr2.tRNA2-TyrGTA	chr2.tRNA3-AlaAGC		
N32I	chr2	43037485	43038007	523	43037732	104	131.08	31.90	125.20	chr2.tRNA5-IleTAT			
N32I	chr2	47562371	47562624	254	47562423	22	5.87	2.93	1.60	BCYRN1			
N32I	chr2	70475952	70476418	467	70476180	78	71.10	15.83	65.83	chr2.tRNA27-GlyCCC			

POLR1C variant	Chr	Start	End	Length	Abs summit	Pileup	-LOG10 (p-value)	FE	-LOG10 (q-value)	First Gene Full Name	Second Gene Full Name	Third Gene Full Name	Fourth Gene Full Name
N32I	chr2	131094536	131094994	459	131094752	50	48.74	15.66	43.80	chr2.tRNA20-GluTTC			
N32I	chr2	157257160	157257930	771	157257680	68	66.94	17.41	61.73	chr2.tRNA13-AlaCGC	chr2.tRNA19-GlyGCC		
N32I	chr3	45730297	45730791	495	45730511	70	69.34	17.80	64.09	chr3.tRNA11-ArgACG			
N32I	chr3	169489778	169490247	470	169490025	81	67.51	13.67	62.29	chr3.tRNA2-ValAAC			
N32I	chr5	140090657	140091205	549	140090903	74	65.95	15.11	60.76	VTRNA1-1			
N32I	chr5	140098280	140098878	599	140098547	95	113.11	27.69	107.42	VTRNA1-2			
N32I	chr5	140105545	140106142	598	140105834	106	116.42	24.29	110.69	VTRNA1-3			
N32I	chr5	180614435	180616113	1679	180615901	78	53.19	9.98	48.19	chr5.tRNA7-LeuAAG	chr5.tRNA15-ValAAC	chr5.tRNA14-ProTGG	
N32I	chr5	180618401	180619009	609	180618719	87	93.57	22.14	88.08	chr5.tRNA13-ThrTGT			
N32I	chr5	180633601	180634146	546	180633882	89	70.71	12.96	65.45	chr5.tRNA8-AlaTGC			
N32I	chr5	180634509	180635079	571	180634808	68	71.75	19.88	66.48	chr5.tRNA9-LysCTT			
N32I	chr5	180644985	180645534	550	180645322	74	74.90	18.88	69.58	chr5.tRNA12-ValAAC			
N32I	chr5	180648761	180649676	916	180649018	70	74.65	20.50	69.34	chr5.tRNA11-LysCTT	chr5.tRNA10-ValCAC		
N32I	chr6	26286550	26286994	445	26286770	88	84.35	17.86	78.94	chr6.tRNA2-MetCAT			
N32I	chr6	26311323	26312283	961	26312041	56	50.26	14.17	45.31	chr6.tRNA174-GlnTTG	chr6.tRNA173-GlnTTG		
N32I	chr6	26312629	26313625	997	26313403	64	61.10	16.31	55.98	chr6.tRNA172-SerTGA	chr6.tRNA171-MetCAT		
N32I	chr6	26322855	26323453	599	26323104	62	57.09	15.34	52.04	chr6.tRNA4-ArgTCG			
N32I	chr6	26327593	26328626	1034	26328433	75	61.64	13.17	56.51	chr6.tRNA5-SerAGA	chr6.tRNA6-ArgACG		
N32I	chr6	26330304	26330843	540	26330532	65	48.74	11.14	43.80	chr6.tRNA169-MetCAT			
N32I	chr6	26331512	26331915	404	26331710	54	41.70	11.20	36.86	chr6.tRNA168-TrpCCA			
N32I	chr6	26521347	26521736	390	26521532	62	61.37	17.33	56.25	chr6.tRNA7-LeuCAG			
N32I	chr6	26532925	26533406	482	26533182	93	75.47	13.48	70.15	chr6.tRNA167-ThrAGT			
N32I	chr6	26537520	26538584	1065	26537754	64	59.52	15.60	54.42	chr6.tRNA8-ArgACG	chr6.tRNA9-ValCAC		
N32I	chr6	26553557	26554638	1082	26554370	79	73.45	16.53	68.15	chr6.tRNA10-AlaCGC	chr6.tRNA11-IleAAT		
N32I	chr6	26555285	26555725	441	26555537	52	39.43	10.76	34.63	chr6.tRNA12-ProAGG			
N32I	chr6	26556594	26557226	633	26556804	68	62.85	15.62	57.70	chr6.tRNA13-LysCTT			
N32I	chr6	26568886	26569353	468	26569118	75	72.16	17.42	66.87	chr6.tRNA14-TyrGTA			
N32I	chr6	26571798	26572387	590	26572129	80	81.30	19.40	75.92	chr6.tRNA166-AlaAGC			

POLR1C variant	Chr	Start	End	Length	Abs summit	Pileup	-LOG10 (p-value)	FE	-LOG10 (q-value)	First Gene Full Name	Second Gene Full Name	Third Gene Full Name	Fourth Gene Full Name
N32I	chr6	26577263	26577646	384	26577374	36	22.18	7.28	17.63	chr6.tRNA16-TyrGTA			
N32I	chr6	26735436	26735757	322	26735635	36	21.79	7.13	17.25	chr6.tRNA164-MetCAT			
N32I	chr6	26745140	26745473	334	26745253	28	16.82	6.58	12.35	chr6.tRNA163-IleAAT			
N32I	chr6	26758464	26758713	250	26758517	20	10.22	5.13	5.86	chr6.tRNA162-MetCAT			
N32I	chr6	26766263	26766684	422	26766440	42	24.84	7.28	20.26	chr6.tRNA27-MetCAT			
N32I	chr6	26987932	26988362	431	26988104	35	22.88	7.84	18.32	chr6.tRNA29-IleTAT			
N32I	chr6	27059293	27059872	580	27059542	52	44.28	12.84	39.41	chr6.tRNA30-ProCGG			
N32I	chr6	27125821	27126199	379	27125997	37	26.91	9.21	22.30	chr6.tRNA33-HisGTG			
N32I	chr6	27129881	27130317	437	27130102	36	24.82	8.58	20.24	chr6.tRNA34-ThrAGT			
N32I	chr6	27144794	27145275	482	27144993	83	58.53	10.53	53.45	chr6.tRNA158-IleAAT			
N32I	chr6	27181457	27181918	462	27181676	52	51.46	16.32	46.49	chr6.tRNA156-ArgACG			
N32I	chr6	27182778	27183219	442	27182994	49	41.90	12.55	37.07	chr6.tRNA36-ArgACG			
N32I	chr6	27198102	27198582	481	27198334	49	36.08	10.04	31.33	chr6.tRNA155-LeuTAA			
N32I	chr6	27203170	27203436	267	27203345	20	10.73	5.39	6.35	chr6.tRNA37-ValAAC			
N32I	chr6	27247859	27248334	476	27248084	60	52.59	13.81	47.60	chr6.tRNA152-ValCAC			
N32I	chr6	27261610	27261840	231	27261729	20	10.93	5.50	6.55	chr6.tRNA41-PseudoACT			
N32I	chr6	27263116	27263456	341	27263256	41	32.33	10.54	27.63	chr6.tRNA42-GlnCTG			
N32I	chr6	27265624	27265922	299	27265851	29	17.09	6.60	12.62	chr6.tRNA43-SerGCT			
N32I	chr6	27446410	27446949	540	27446657	65	54.71	13.37	49.69	chr6.tRNA44-SerAGA			
N32I	chr6	27447295	27447749	455	27447518	52	45.62	13.28	40.73	chr6.tRNA45-AspGTC			
N32I	chr6	27463415	27463865	451	27463628	40	30.21	9.91	25.54	chr6.tRNA46-SerAGA			
N32I	chr6	27470656	27471131	476	27470875	58	46.33	11.92	41.43	chr6.tRNA47-SerAGA			
N32I	chr6	27471420	27471755	336	27471578	39	22.07	6.80	17.52	chr6.tRNA48-AspGTC			
N32I	chr6	27473488	27473737	250	27473635	27	15.27	6.13	10.82	chr6.tRNA148-SerTGA			
N32I	chr6	27487108	27487601	494	27487324	83	63.73	12.03	58.57	chr6.tRNA49-GlnCTG			
N32I	chr6	27515349	27515805	457	27515537	73	57.94	12.47	52.87	chr6.tRNA146-GlnCTG			
N32I	chr6	27543818	27544058	241	27543928	20	12.07	6.11	7.67	chr6.tRNA53-LysTTT			

POLR1C variant	Chr	Start	End	Length	Abs summit	Pileup	-LOG10 (p-value)	FE	-LOG10 (q-value)	First Gene Full Name	Second Gene Full Name	Third Gene Full Name	Fourth Gene Full Name
N32I	chr6	27551099	27551519	421	27551304	60	59.73	17.07	54.64	chr6.tRNA144-AspGTC			
N32I	chr6	27559448	27559789	342	27559616	34	24.75	8.83	20.17	chr6.tRNA143-LysTTT			
N32I	chr6	27560411	27560869	459	27560624	79	68.09	14.50	62.87	chr6.tRNA142-MetCAT			
N32I	chr6	27585956	27586230	275	27586107	21	11.80	5.68	7.40	chr6.tRNA54-ThrCGT			
N32I	chr6	27618458	27618959	502	27618753	51	44.37	13.09	39.50	chr6.tRNA139-ValAAC			
N32I	chr6	27636255	27636661	407	27636460	38	32.84	11.75	28.14	chr6.tRNA57-IleAAT			
N32I	chr6	27640188	27640444	257	27640352	18	10.09	5.40	5.72	chr6.tRNA137-SerCGA			
N32I	chr6	27652282	27652702	421	27652475	41	21.01	6.14	16.48	chr6.tRNA135-ThrAGT			
N32I	chr6	27655869	27656308	440	27656088	49	28.13	7.28	23.49	chr6.tRNA59-IleAAT			
N32I	chr6	27720950	27721460	511	27721256	73	64.68	14.82	59.51	chr6.tRNA132-ValAAC			
N32I	chr6	27758919	27759403	485	27759183	73	47.98	9.26	43.06	chr6.tRNA131-GlnCTG			
N32I	chr6	27763441	27763978	538	27763667	83	85.55	20.09	80.13	chr6.tRNA130-GlnTTG			
N32I	chr6	27870057	27870987	931	27870732	86	86.61	19.57	81.18	chr6.tRNA129-MetCAT	chr6.tRNA128-GlyGCC		
N32I	chr6	28180578	28181111	534	28180858	89	84.75	17.78	79.33	chr6.tRNA62-SerGCT			
N32I	chr6	28442064	28442597	534	28442352	80	51.04	9.04	46.08	chr6.tRNA127-ThrTGT			
N32I	chr6	28456604	28457021	418	28456817	56	31.42	7.18	26.74	chr6.tRNA125-ThrCGT			
N32I	chr6	28510698	28511157	460	28510928	57	44.56	11.40	39.68	chr6.tRNA124-ArgTCG			
N32I	chr6	28556970	28557484	515	28557185	61	44.22	10.39	39.35	chr6.tRNA64-GlnTTG			
N32I	chr6	28564908	28565393	486	28565185	57	39.89	9.79	35.08	chr6.tRNA123-SerGCT			
N32I	chr6	28574809	28575186	378	28574985	52	30.94	7.59	26.27	chr6.tRNA65-AlaAGC			
N32I	chr6	28611013	28611498	486	28611245	50	43.13	12.91	38.28	chr6.tRNA66-AlaTGC			
N32I	chr6	28615777	28616266	490	28616047	70	54.44	11.99	49.43	chr6.tRNA121-ThrCGT			
N32I	chr6	28625811	28626349	539	28626055	78	71.10	15.83	65.83	chr6.tRNA120-AlaAGC			
N32I	chr6	28641414	28641961	548	28641659	79	82.38	20.33	76.98	chr6.tRNA119-AlaCGC			
N32I	chr6	28696892	28697341	450	28697138	61	47.87	11.72	42.95	chr6.tRNA70-AlaCGC			
N32I	chr6	28703070	28703447	378	28703266	24	14.51	6.40	10.08	chr6.tRNA115-ValAAC			
N32I	chr6	28715333	28715788	456	28715534	54	41.13	10.83	36.30	chr6.tRNA71-LysTTT			

POLR1C variant	Chr	Start	End	Length	Abs summit	Pileup	-LOG10 (p-value)	FE	-LOG10 (q-value)	First Gene Full Name	Second Gene Full Name	Third Gene Full Name	Fourth Gene Full Name
N32I	chr6	28726033	28726373	341	28726219	29	15.14	5.75	10.70	chr6.tRNA113-AlaTGC			
N32I	chr6	28757384	28757706	323	28757589	41	27.56	8.45	22.93	chr6.tRNA110-AlaTGC			
N32I	chr6	28758367	28758707	341	28758517	28	20.01	8.12	15.49	chr6.tRNA109-PheGAA			
N32I	chr6	28763491	28764032	542	28763787	74	72.96	18.02	67.67	chr6.tRNA108-AlaAGC			
N32I	chr6	28770391	28770804	414	28770607	36	22.55	7.44	18.00	chr6.tRNA107-AlaTGC			
N32I	chr6	28775466	28775993	528	28775635	41	32.38	10.56	27.68	chr6.tRNA106-PheGAA			
N32I	chr6	28784844	28785227	384	28785048	36	19.39	6.32	14.88	chr6.tRNA104-AlaTGC			
N32I	chr6	28806006	28806465	460	28806253	78	71.10	15.83	65.83	chr6.tRNA102-AlaAGC			
N32I	chr6	28831275	28831759	485	28831507	69	70.65	18.86	65.39	chr6.tRNA101-AlaAGC			
N32I	chr6	28848915	28849374	460	28849157	62	60.07	16.52	54.97	chr6.tRNA73-ArgCCG			
N32I	chr6	28863747	28864327	581	28864076	98	110.02	24.85	104.37	chr6.tRNA100-LeuCAA			
N32I	chr6	28908620	28909604	985	28908869	102	103.70	20.75	98.12	chr6.tRNA74-LeuCAA	chr6.tRNA99-GlnCTG		
N32I	chr6	28911236	28911685	450	28911436	47	39.70	12.12	34.90	chr6.tRNA98-LeuAAG			
N32I	chr6	28912129	28912629	501	28912373	78	80.94	20.15	75.56	chr6.tRNA75-MetCAT			
N32I	chr6	28918560	28919122	563	28918827	91	88.42	18.58	82.97	chr6.tRNA76-LysTTT			
N32I	chr6	28920816	28921331	516	28921080	87	83.00	17.71	77.61	chr6.tRNA97-MetCAT			
N32I	chr6	28949277	28950266	990	28950023	95	109.21	25.83	103.56	chr6.tRNA96-PheGAA	chr6.tRNA77-GluCTC		
N32I	chr6	52860252	52860887	636	52860422	60	46.72	11.45	41.81	RN7SK			
N32I	chr6	126101121	126101699	579	126101464	91	88.42	18.44	82.97	chr6.tRNA87-GluCTC			
N32I	chr6	144537516	144537978	463	144537721	49	43.79	13.44	38.93	chr6.tRNA83-LeuTAA			
N32I	chr7	128423309	128423760	452	128423540	63	52.28	12.94	47.30	chr7.tRNA2-ProAGG			
N32I	chr7	139025245	139025716	472	139025469	76	77.12	19.16	71.78	chr7.tRNA3-ArgCCT			
N32I	chr7	148638381	148638875	495	148638600	81	61.44	11.82	56.32	RNY5			
N32I	chr7	148660158	148660707	550	148660461	94	89.39	17.83	83.93	RNY4			
N32I	chr7	148680620	148681119	500	148680897	53	35.75	9.09	31.00	RNY3			
N32I	chr7	148684016	148684540	525	148684314	78	63.88	13.31	58.72	RNY1			
N32I	chr7	149404616	149404942	327	149404784	31	17.84	6.42	13.35	chr7.tRNA15-CysGCA			
N32I	chr8	67025440	67025835	396	67025630	86	89.84	20.78	84.38	chr8.tRNA4-TyrGTA			
N32I	chr8	67026072	67026665	594	67026440	35	29.53	11.05	24.88	chr8.tRNA5-TyrGTA	chr8.tRNA6-AlaAGC		

POLR1C variant	Chr	Start	End	Length	Abs summit	Pileup	-LOG10 (p-value)	FE	-LOG10 (q-value)	First Gene Full Name	Second Gene Full Name	Third Gene Full Name	Fourth Gene Full Name
N32I	chr8	96281713	96282133	421	96281916	65	58.95	14.96	53.87	chr8.tRNA11-SerAGA			
N32I	chr8	124169259	124169753	495	124169525	67	71.64	20.12	66.36	chr8.tRNA10-MetCAT			
N32I	chr9	35657715	35658118	404	35657894	43	22.73	6.35	18.17	RMRP			
N32I	chr9	131102236	131102490	255	131102356	30	16.93	6.28	12.46	chr9.tRNA4-ArgTCT			
N32I	chr9	137029497	137029809	313	137029634	22	15.06	7.10	10.61	RNU6ATAC			
N32I	chr10	5895506	5895898	393	5895727	30	23.68	9.52	19.11	chr10.tRNA6-ValTAC			
N32I	chr10	22518237	22518729	493	22518461	88	69.54	12.75	64.29	chr10.tRNA4-AsnGTT			
N32I	chr10	69524053	69524603	551	69524314	63	59.85	16.17	54.75	chr10.tRNA2-SerTGA			
N32I	chr11	59317943	59319501	1559	59318132	79	79.90	19.23	74.53	chr11.tRNA17-ValTAC	chr11.tRNA16-ValTAC	chr11.tRNA3-ArgTCT	chr11.tRNA4-LeuTAA
N32I	chr11	59323734	59324173	440	59323917	54	41.13	10.83	36.30	chr11.tRNA5-LysTTT			
N32I	chr11	59324761	59325228	468	59325027	56	54.92	16.37	49.89	chr11.tRNA15-PheGAA			
N32I	chr11	59327590	59328109	520	59327846	91	88.42	18.58	82.97	chr11.tRNA14-LysTTT			
N32I	chr11	59333573	59334153	581	59333862	76	77.71	19.43	72.36	chr11.tRNA13-PheGAA			
N32I	chr11	66115376	66115884	509	66115640	86	92.10	21.78	86.62	chr11.tRNA8-SerGCT			
N32I	chr11	75946312	75947199	888	75946581	112	118.04	22.63	112.29	chr11.tRNA9-ProAGG	chr11.tRNA12-ProTGG		
N32I	chr12	56583987	56584282	296	56584134	26	19.24	8.42	14.74	chr12.tRNA2-SerCGA			
N32I	chr12	96429633	96430073	441	96429828	80	71.05	15.25	65.78	chr12.tRNA4-AspGTC			
N32I	chr12	98897076	98897520	445	98897335	69	71.64	19.33	66.37	chr12.tRNA5-AspGTC			
N32I	chr12	98897883	98898351	469	98898058	60	55.88	15.27	50.84	chr12.tRNA6-TrpCCA			
N32I	chr12	125406110	125406581	472	125406322	86	84.99	18.74	79.57	chr12.tRNA13-AlaTGC			
N32I	chr12	125411684	125412740	1057	125411925	102	105.06	21.13	99.46	chr12.tRNA12-AspGTC	chr12.tRNA11-PheGAA		
N32I	chr12	125423964	125424928	965	125424352	79	75.45	17.34	70.13	chr12.tRNA10-AspGTC	chr12.tRNA8-AlaTGC		
N32I	chr13	31247915	31248286	372	31248105	34	28.34	10.83	23.70	chr13.tRNA7-AsnGTT			
N32I	chr13	41634723	41634989	267	41634863	24	17.11	7.76	12.64	chr13.tRNA5-GluTTC			
N32I	chr13	45491890	45492298	409	45492083	59	61.22	18.29	56.10	chr13.tRNA3-GluTTC			
N32I	chr13	95201525	95202230	706	95201954	101	114.61	25.58	108.89	chr13.tRNA1-PheGAA			
N32I	chr14	21077301	21077782	482	21077542	67	65.23	17.07	60.05	chr14.tRNA23-ProAGG			
N32I	chr14	21078108	21078579	472	21078345	73	73.87	18.87	68.57	chr14.tRNA1-LeuAAG			

POLR1C variant	Chr	Start	End	Length	Abs summit	Pileup	-LOG10 (p-value)	FE	-LOG10 (q-value)	First Gene Full Name	Second Gene Full Name	Third Gene Full Name	Fourth Gene Full Name
N32I	chr14	21081340	21082256	917	21081663	83	69.95	14.04	64.70	chr14.tRNA22-ProAGG	chr14.tRNA21-ThrTGT		
N32I	chr14	21093303	21093884	582	21093565	75	67.23	15.25	62.02	chr14.tRNA2-LeuTAG			
N32I	chr14	21099091	21099561	471	21099345	54	54.21	16.76	49.20	chr14.tRNA20-ThrTGT			
N32I	chr14	21100996	21101587	592	21101207	35	29.53	11.05	24.88	chr14.tRNA3-ProTGG			
N32I	chr14	21149643	21150137	495	21149917	80	74.77	16.68	69.45	chr14.tRNA4-ThrTGT			
N32I	chr14	21151202	21152479	1278	21151464	70	60.90	14.24	55.78	chr14.tRNA5-TyrGTA			
N32I	chr14	23398676	23399260	585	23398955	107	109.66	21.32	104.01	chr14.tRNA7-ArgACG			
N32I	chr14	32672189	32672667	479	32672404	41	36.95	12.81	32.18	RNU6-8			
N32I	chr14	50053081	50053723	643	50053488	39	22.07	6.68	17.52	RN7SL1			
N32I	chr14	50329092	50329693	602	50329364	38	28.97	9.84	24.32	RN7SL2			
N32I	chr14	58706389	58706895	507	58706645	82	57.46	10.44	52.39	chr14.tRNA13-LysCTT			
N32I	chr14	73429567	73429930	364	73429741	30	23.68	9.52	19.11	chr14.tRNA8-CysGCA			
N32I	chr14	102783244	102783667	424	102783455	60	55.88	15.27	50.84	chr14.tRNA10-IleAAT			
N32I	chr15	40885767	40886320	554	40886095	77	87.85	23.78	82.41	chr15.tRNA10-SerGCT			
N32I	chr15	45490583	45491066	484	45490867	56	50.69	14.36	45.73	chr15.tRNA9-HisGTG			
N32I	chr15	45492400	45493620	1221	45492631	52	51.46	16.10	46.49	chr15.tRNA8-HisGTG	chr15.tRNA1-HisGTG		
N32I	chr15	66161186	66161705	520	66161456	47	39.47	12.19	34.67	chr15.tRNA7-GlnCTG			
N32I	chr15	68132253	68132512	260	68132436	23	13.59	6.04	9.16	RNU6-1			
N32I	chr15	79152727	79153152	426	79152937	62	58.52	15.81	53.44	chr15.tRNA2-LysCTT			
N32I	chr15	89878041	89878647	607	89878344	134	146.34	25.43	140.29	chr15.tRNA4-ArgTCG			
N32I	chr16	686472	686974	503	686760	50	29.06	7.38	24.41	chr16.tRNA34-GlyCCC			
N32I	chr16	3200515	3200934	420	3200720	54	41.70	11.05	36.86	chr16.tRNA1-ArgCCG			
N32I	chr16	3202704	3203277	574	3203018	61	57.20	15.63	52.14	chr16.tRNA2-ArgCCT			
N32I	chr16	3207247	3207702	456	3207435	54	41.70	11.20	36.86	chr16.tRNA32-LysCTT			
N32I	chr16	3232539	3232773	235	3232709	24	9.80	4.27	5.44	chr16.tRNA29-ProAGG			
N32I	chr16	3239482	3239815	334	3239660	28	21.43	8.86	16.89	chr16.tRNA9-ProAGG			
N32I	chr16	14379552	14380041	490	14379816	60	62.64	18.51	57.50	chr16.tRNA15-ThrCGT			
N32I	chr16	22206771	22207332	562	22207079	62	45.40	10.66	40.51	chr16.tRNA27-LeuTAG			
N32I	chr16	22308278	22308759	482	22308498	61	49.88	12.50	44.93	chr16.tRNA16-LeuAAG			

POLR1C variant	Chr	Start	End	Length	Abs summit	Pileup	-LOG10 (p-value)	FE	-LOG10 (q-value)	First Gene Full Name	Second Gene Full Name	Third Gene Full Name	Fourth Gene Full Name
N32I	chr16	57333633	57334679	1047	57334428	89	96.52	22.50	91.00	chr16.tRNA17-LeuCAG	chr16.tRNA26-LeuCAG		
N32I	chr16	70812728	70813155	428	70812980	40	35.68	12.59	30.94	chr16.tRNA24-GlyGCC			
N32I	chr16	87417485	87417895	411	87417668	49	47.39	15.22	42.48	chr16.tRNA22-MetCAT			
N32I	chr17	8022281	8024427	2147	8023095	70	49.25	10.17	44.31	chr17.tRNA2-LysTTT	chr17.tRNA3-GlnCTG	chr17.tRNA42-LeuTAG	
N32I	chr17	8028879	8029278	400	8029110	41	32.38	10.74	27.68	chr17.tRNA5-GlyGCC			
N32I	chr17	8041992	8043005	1014	8042204	38	33.19	11.93	28.48	chr17.tRNA41-SerCGA	chr17.tRNA40-ThrAGT		
N32I	chr17	8089498	8091232	1735	8090243	65	62.86	16.68	57.71	chr17.tRNA6-TrpCCA	chr17.tRNA7-SerGCT	chr17.tRNA8-ThrAGT	chr17.tRNA9-IleAAT
N32I	chr17	8123964	8125079	1116	8124222	60	55.88	15.27	50.84	chr17.tRNA39-TrpCCA	chr17.tRNA10-GlyTCC		
N32I	chr17	8125362	8126389	1028	8125547	51	44.63	13.21	39.76	chr17.tRNA38-AspGTC	chr17.tRNA37-ProCGG		
N32I	chr17	8129460	8129717	258	8129595	25	18.17	7.98	13.68	chr17.tRNA36-ThrAGT			
N32I	chr17	8130046	8130407	362	8130248	23	16.08	7.32	11.61	chr17.tRNA34-IleAAT			
N32I	chr17	19411243	19411750	508	19411513	82	76.34	16.70	71.01	chr17.tRNA12-TrpCCA			
N32I	chr17	29876948	29877453	506	29877165	49	47.39	15.44	42.48	chr17.tRNA14-ThrCGT			
N32I	chr17	36907872	36908333	462	36908090	47	44.73	14.56	39.85	chr17.tRNA31-AsnGTT			
N32I	chr17	37023702	37024167	466	37023955	68	66.60	17.26	61.39	chr17.tRNA15-CysGCA			
N32I	chr17	37025400	37025747	348	37025599	49	39.24	11.52	34.44	chr17.tRNA28-CysGCA			
N32I	chr17	37309761	37311036	1276	37310778	94	103.98	23.77	98.39	chr17.tRNA27-CysGCA	chr17.tRNA26-CysGCA		
N32I	chr17	47269647	47270239	593	47269915	89	101.95	25.01	96.39	chr17.tRNA16-GlnTTG			
N32I	chr17	66015749	66016301	553	66016053	95	114.24	28.25	108.53	chr17.tRNA23-ArgCCG			
N32I	chr17	73029849	73031477	1629	73031273	70	69.70	17.96	64.45	chr17.tRNA18-ArgCCT	chr17.tRNA21-ArgCCT	chr17.tRNA19-ArgTCG	
N32I	chr17	80452279	80452925	647	80452647	102	74.42	11.45	69.11	chr17.tRNA20-MetCAT			
N32I	chr19	893274	893685	412	893522	54	54.21	16.98	49.20	RNU6-9			
N32I	chr19	1021323	1021907	585	1021549	94	65.66	10.56	60.47	RNU6-2			
N32I	chr19	1383156	1383827	672	1383481	50	43.13	12.91	38.28	chr19.tRNA14-PheGAA			
N32I	chr19	4723884	4724901	1018	4724125	76	86.32	23.56	80.89	chr19.tRNA2-GlyTCC	chr19.tRNA13-ValCAC		
N32I	chr19	33667768	33668289	522	33668055	41	36.95	12.81	32.18	chr19.tRNA4-ThrAGT			
N32I	chr19	45981697	45982156	460	45981892	34	28.34	10.62	23.70	chr19.tRNA8-SeCTCA			
N32I	chr21	18826888	18827409	522	18827107	65	69.87	20.27	64.61	chr21.tRNA2-GlyGCC			

POLR1C variant	Chr	Start	End	Length	Abs summit	Pileup	-LOG10 (p-value)	FE	-LOG10 (q-value)	First Gene Full Name	Second Gene Full Name	Third Gene Full Name	Fourth Gene Full Name
N32I	chrX	18692871	18693258	388	18693053	36	30.74	11.27	26.06	chrX.tRNA4-ValTAC			
N74S	chr1	16861735	16862006	272	16861818	14	6.53	3.98	2.22	chr1.tRNA134-GluTTC			
N74S	chr1	16872215	16872752	538	16872508	59	47.50	12.08	42.35	chr1.tRNA133-GlyCCC			
N74S	chr1	16873991	16874291	301	16874232	25	15.45	6.61	10.90	chr1.tRNA132-PseudoCAC			
N74S	chr1	17004561	17005053	493	17004763	46	36.78	11.12	31.82	chr1.tRNA131-GlyCCC			
N74S	chr1	17053692	17054054	363	17053852	48	27.27	7.03	22.47	chr1.tRNA2-GlyCCC			
N74S	chr1	17186595	17186879	285	17186733	25	15.45	6.61	10.90	chr1.tRNA3-PseudoCAC			
N74S	chr1	17188299	17188695	397	17188460	49	40.03	11.66	35.01	chr1.tRNA4-GlyCCC			
N74S	chr1	94312889	94313393	505	94313167	64	53.49	13.02	48.24	chr1.tRNA9-ArgTCT			
N74S	chr1	145395330	145395817	488	145395577	69	67.96	17.69	62.50	chr1.tRNA119-LysCTT			
N74S	chr1	145396772	145397131	360	145396930	36	26.35	9.25	21.58	chr1.tRNA118-HisGTG			
N74S	chr1	145397609	145398202	594	145397919	84	78.99	17.17	73.45	chr1.tRNA117-GlyTCC			
N74S	chr1	145398985	145399488	504	145399231	113	135.75	29.63	129.93	chr1.tRNA116-GluCTC			
N74S	chr1	148248122	148248370	249	148248313	16	5.95	3.41	1.67	chr1.tRNA26-AsnGTT			
N74S	chr1	148598201	148598639	439	148598393	32	21.26	7.71	16.59	chr1.tRNA108-AsnGTT			
N74S	chr1	148760092	148760585	494	148760320	31	20.17	7.45	15.52	chr1.tRNA107-AsnGTT			
N74S	chr1	149298316	149298672	357	149298576	44	22.17	6.02	17.48	chr1.tRNA98-ValCAC			
N74S	chr1	149679989	149680487	499	149680279	81	65.62	13.05	60.18	chr1.tRNA91-PseudoCCC			
N74S	chr1	149683918	149684325	408	149684169	71	42.19	8.07	37.13	chr1.tRNA90-ValCAC			
N74S	chr1	153643490	153644003	514	153643753	97	97.62	19.98	91.97	chr1.tRNA32-MetCAT			
N74S	chr1	161369219	161369780	562	161369519	64	61.19	16.28	55.81	chr1.tRNA85-ValCAC			
N74S	chr1	161391703	161392056	354	161391897	23	14.36	6.53	9.82	chr1.tRNA84-GluTTC			
N74S	chr1	161397698	161398130	433	161397921	48	44.11	13.90	39.01	chr1.tRNA83-AsnGTT			
N74S	chr1	161412886	161413410	525	161413139	73	47.44	9.17	42.29	chr1.tRNA35-GlyGCC			
N74S	chr1	161416792	161417351	560	161417005	94	34.69	4.76	29.77	chr1.tRNA80-GluCTC			
N74S	chr1	161420308	161420725	418	161420495	93	57.60	8.85	52.27	chr1.tRNA37-GlyGCC			
N74S	chr1	161424195	161424761	567	161424434	132	86.01	9.98	80.42	chr1.tRNA77-GluCTC			

POLR1C variant	Chr	Start	End	Length	Abs summit	Pileup	-LOG10 (p-value)	FE	-LOG10 (q-value)	First Gene Full Name	Second Gene Full Name	Third Gene Full Name	Fourth Gene Full Name
N74S	chr1	161425239	161425515	277	161425390	43	16.30	4.45	11.73	chr1.tRNA75-AspGTC			
N74S	chr1	161427763	161428125	363	161427955	68	33.20	6.28	28.31	chr1.tRNA39-GlyGCC			
N74S	chr1	161431588	161432160	573	161431835	99	66.41	10.09	60.96	chr1.tRNA74-GluCTC			
N74S	chr1	161435138	161435525	388	161435352	73	32.80	5.73	27.91	chr1.tRNA41-GlyGCC			
N74S	chr1	161438969	161439531	563	161439219	120	71.72	8.69	66.22	chr1.tRNA71-GluCTC			
N74S	chr1	161493454	161493883	430	161493671	60	43.20	10.22	38.12	chr1.tRNA68-GlyGCC			
N74S	chr1	161499900	161500406	507	161500167	74	72.69	17.89	67.18	chr1.tRNA67-LeuCAG			
N74S	chr1	161500711	161501155	445	161500922	55	37.84	9.44	32.86	chr1.tRNA45-GlyTCC			
N74S	chr1	161509874	161510171	298	161510071	45	27.50	7.63	22.70	chr1.tRNA47-AsnGTT			
N74S	chr1	161582285	161582773	489	161582533	42	35.29	11.69	30.36	chr1.tRNA49-GluTTC			
N74S	chr1	167683781	167684238	458	167684003	35	29.01	10.89	24.18	chr1.tRNA52-ProCGG			
N74S	chr1	167684576	167684972	397	167684811	41	36.33	12.56	31.37	chr1.tRNA65-ProAGG			
N74S	chr1	204475493	204476546	1054	204476199	91	88.42	18.49	82.81	chr1.tRNA54-LysTTT	chr1.tRNA62-LysTTT		
N74S	chr1	222638123	222638591	469	222638398	44	37.41	12.05	32.44	chr1.tRNA56-ThrTGT			
N74S	chr1	228745885	228746207	323	228746014	62	21.02	4.21	16.35	RNA5S1			
N74S	chr1	228748118	228748478	361	228748292	82	43.36	7.10	38.28	RNA5S2			
N74S	chr1	228750334	228750769	436	228750547	81	41.00	6.64	35.96	RNA5S3			
N74S	chr1	228752549	228752900	352	228752777	55	12.21	2.98	7.72	RNA5S4			
N74S	chr1	228754853	228755172	320	228755003	66	20.72	4.00	16.06	RNA5S5			
N74S	chr1	228756985	228757367	383	228757192	74	27.61	4.72	22.81	RNA5S6			
N74S	chr1	228759263	228759580	318	228759436	67	26.20	4.92	21.43	RNA5S7			
N74S	chr1	228761477	228761878	402	228761715	67	27.76	5.22	22.96	RNA5S8			
N74S	chr1	228763765	228764060	296	228763863	50	18.52	4.50	13.90	RNA5S9			
N74S	chr1	228766011	228766349	339	228766185	62	28.19	5.66	23.37	RNA5S10			
N74S	chr1	228768277	228768605	329	228768403	69	25.83	4.72	21.06	RNA5S11			
N74S	chr1	228770517	228770845	329	228770672	72	35.69	6.42	30.75	RNA5S12			
N74S	chr1	228772799	228773052	254	228772908	62	28.29	5.69	23.48	RNA5S13			
N74S	chr1	228774952	228775302	351	228775114	68	31.08	5.82	26.23	RNA5S14			
N74S	chr1	228781626	228781992	367	228781787	62	18.26	3.72	13.65	RNA5S17			
N74S	chr1	249167893	249168695	803	249168431	56	50.69	14.39	45.48	chr1.tRNA58-LeuCAA	chr1.tRNA59-GluCTC		
N74S	chr2	27273427	27274310	884	27273685	98	98.09	19.81	92.43	chr2.tRNA2-TyrGTA	chr2.tRNA3-AlaAGC		
N74S	chr2	43037484	43037984	501	43037720	64	63.44	17.32	58.02	chr2.tRNA5-IleTAT			
N74S	chr2	70475917	70476384	468	70476160	68	66.60	17.46	61.14	chr2.tRNA27-GlyCCC			
N74S	chr2	131094546	131094930	385	131094733	36	26.80	9.47	22.01	chr2.tRNA20-GluTTC			

POLR1C variant	Chr	Start	End	Length	Abs summit	Pileup	-LOG10 (p-value)	FE	-LOG10 (q-value)	First Gene Full Name	Second Gene Full Name	Third Gene Full Name	Fourth Gene Full Name
N74S	chr2	157257101	157258029	929	157257734	51	44.37	13.21	39.27	chr2.tRNA13-AlaCGC	chr2.tRNA19-GlyGCC		
N74S	chr3	45730287	45730762	476	45730509	57	45.16	11.70	40.04	chr3.tRNA11-ArgACG			
N74S	chr3	169489850	169490269	420	169490037	69	50.50	10.85	45.30	chr3.tRNA2-ValAAC			
N74S	chr5	140090674	140091177	504	140090937	61	57.20	15.57	51.88	VTRNA1-1			
N74S	chr5	140098323	140098780	458	140098536	84	78.99	17.17	73.45	VTRNA1-2			
N74S	chr5	140105522	140106006	485	140105778	80	86.23	21.58	80.64	VTRNA1-3			
N74S	chr5	180614495	180614942	448	180614716	63	59.85	16.04	54.49	chr5.tRNA7-LeuAAG			
N74S	chr5	180615231	180616126	896	180615494	59	54.57	15.10	49.30	chr5.tRNA15-ValAAC	chr5.tRNA14-ProTGG		
N74S	chr5	180618501	180618948	448	180618763	53	49.87	15.11	44.68	chr5.tRNA13-ThrTGT			
N74S	chr5	180633693	180634183	491	180633905	61	49.88	12.46	44.68	chr5.tRNA8-AlaTGC			
N74S	chr5	180634608	180635061	454	180634842	56	49.03	13.64	43.85	chr5.tRNA9-LysCTT			
N74S	chr5	180645003	180645601	599	180645281	59	60.32	17.86	54.95	chr5.tRNA12-ValAAC			
N74S	chr5	180648737	180649614	878	180649438	50	40.23	11.67	35.21	chr5.tRNA11-LysCTT	chr5.tRNA10-ValCAC		
N74S	chr6	26286572	26287064	493	26286793	65	65.75	18.26	60.31	chr6.tRNA2-MetCAT			
N74S	chr6	26311798	26312239	442	26312013	34	24.00	8.69	19.27	chr6.tRNA173-GlnTTG			
N74S	chr6	26312708	26313616	909	26313404	63	60.14	16.17	54.78	chr6.tRNA172-SerTGA	chr6.tRNA171-MetCAT		
N74S	chr6	26322869	26323341	473	26323093	67	65.77	17.46	60.32	chr6.tRNA4-ArgTCG			
N74S	chr6	26327594	26328629	1036	26327816	62	58.41	15.75	53.07	chr6.tRNA5-SerAGA	chr6.tRNA6-ArgACG		
N74S	chr6	26330320	26330838	519	26330539	68	65.40	16.91	59.96	chr6.tRNA169-MetCAT			
N74S	chr6	26331576	26331973	398	26331717	32	13.78	4.86	9.25	chr6.tRNA168-TrpCCA			
N74S	chr6	26521376	26521746	371	26521552	60	55.39	15.11	50.10	chr6.tRNA7-LeuCAG			
N74S	chr6	26532905	26533477	573	26533162	75	73.61	17.91	68.10	chr6.tRNA167-ThrAGT			
N74S	chr6	26537553	26538432	880	26537807	61	39.81	8.90	34.80	chr6.tRNA8-ArgACG			
N74S	chr6	26553510	26554647	1138	26553744	65	54.71	13.40	49.43	chr6.tRNA10-AlaCGC	chr6.tRNA11-IleAAT		
N74S	chr6	26555371	26555832	462	26555610	43	25.79	7.39	21.02	chr6.tRNA12-ProAGG			
N74S	chr6	26556588	26557095	508	26556794	43	33.07	10.34	28.18	chr6.tRNA13-LysCTT			
N74S	chr6	26568868	26569316	449	26569106	74	60.17	12.95	54.81	chr6.tRNA14-TyrGTA			
N74S	chr6	26571857	26572333	477	26572133	55	45.60	12.47	40.48	chr6.tRNA166-AlaAGC			

POLR1C variant	Chr	Start	End	Length	Abs summit	Pileup	-LOG10 (p-value)	FE	-LOG10 (q-value)	First Gene Full Name	Second Gene Full Name	Third Gene Full Name	Fourth Gene Full Name
N74S	chr6	26577218	26577561	344	26577372	45	36.29	11.20	31.34	chr6.tRNA16-TyrGTA			
N74S	chr6	26735441	26735792	352	26735618	27	14.17	5.61	9.64	chr6.tRNA164-MetCAT			
N74S	chr6	26745099	26745405	307	26745260	31	17.76	6.39	13.16	chr6.tRNA163-IleAAT			
N74S	chr6	26766281	26766722	442	26766472	39	23.35	7.25	18.63	chr6.tRNA27-MetCAT			
N74S	chr6	27059288	27059756	469	27059509	44	34.21	10.56	29.30	chr6.tRNA30-ProCGG			
N74S	chr6	27125860	27126174	315	27126021	29	18.37	7.09	13.76	chr6.tRNA33-HisGTG			
N74S	chr6	27129949	27130247	299	27130094	29	17.74	6.79	13.14	chr6.tRNA34-ThrAGT			
N74S	chr6	27144766	27145286	521	27145022	52	39.43	10.76	34.42	chr6.tRNA158-IleAAT			
N74S	chr6	27181487	27181859	373	27181666	49	45.97	14.73	40.84	chr6.tRNA156-ArgACG			
N74S	chr6	27182786	27183148	363	27182950	37	29.78	10.62	24.95	chr6.tRNA36-ArgACG			
N74S	chr6	27198214	27198666	453	27198432	42	30.01	9.28	25.17	chr6.tRNA155-LeuTAA			
N74S	chr6	27247868	27248228	361	27248047	53	35.75	9.12	30.81	chr6.tRNA152-ValCAC			
N74S	chr6	27265662	27265949	288	27265810	31	20.52	7.61	15.86	chr6.tRNA43-SerGCT			
N74S	chr6	27446435	27447679	1245	27446637	56	50.77	14.43	45.56	chr6.tRNA44-SerAGA	chr6.tRNA45-AspGTC		
N74S	chr6	27463384	27463830	447	27463621	39	29.90	10.06	25.07	chr6.tRNA46-SerAGA			
N74S	chr6	27470685	27470984	300	27470847	30	18.53	6.93	13.91	chr6.tRNA47-SerAGA			
N74S	chr6	27471372	27471786	415	27471530	25	13.68	5.77	9.15	chr6.tRNA48-AspGTC			
N74S	chr6	27473459	27473760	302	27473599	32	18.75	6.80	14.13	chr6.tRNA148-SerTGA			
N74S	chr6	27487117	27487606	490	27487300	51	42.27	12.26	37.21	chr6.tRNA49-GlnCTG			
N74S	chr6	27515326	27515787	462	27515568	69	68.29	17.84	62.82	chr6.tRNA146-GlnCTG			
N74S	chr6	27551062	27551465	404	27551261	41	23.91	7.08	19.18	chr6.tRNA144-AspGTC			
N74S	chr6	27559466	27559802	337	27559640	31	21.41	8.03	16.74	chr6.tRNA143-LysTTT			
N74S	chr6	27560317	27560930	614	27560657	59	58.36	16.89	53.03	chr6.tRNA142-MetCAT			
N74S	chr6	27618546	27618963	418	27618776	41	32.38	10.62	27.50	chr6.tRNA139-ValAAC			
N74S	chr6	27636227	27636645	419	27636436	42	33.54	10.86	28.64	chr6.tRNA57-IleAAT			
N74S	chr6	27652349	27652716	368	27652455	38	32.62	11.72	27.73	chr6.tRNA135-ThrAGT			
N74S	chr6	27655779	27656297	519	27656044	40	33.47	11.47	28.58	chr6.tRNA59-IleAAT			

POLR1C variant	Chr	Start	End	Length	Abs summit	Pileup	-LOG10 (p-value)	FE	-LOG10 (q-value)	First Gene Full Name	Second Gene Full Name	Third Gene Full Name	Fourth Gene Full Name
N74S	chr6	27720975	27721477	503	27721224	73	76.33	19.95	70.81	chr6.tRNA132-ValAAC			
N74S	chr6	27758889	27759331	443	27759139	56	44.52	11.72	39.42	chr6.tRNA131-GlnCTG			
N74S	chr6	27763422	27763911	490	27763704	67	50.68	11.37	45.47	chr6.tRNA130-GlnTTG			
N74S	chr6	27870083	27871029	947	27870309	68	68.99	18.57	63.51	chr6.tRNA129-MetCAT	chr6.tRNA128-GlyGCC		
N74S	chr6	28180591	28181082	492	28180853	75	54.71	10.92	49.43	chr6.tRNA62-SerGCT			
N74S	chr6	28442150	28442607	458	28442378	69	65.91	16.77	60.46	chr6.tRNA127-ThrTGT			
N74S	chr6	28456666	28457020	355	28456775	43	20.21	5.55	15.56	chr6.tRNA125-ThrCGT			
N74S	chr6	28510750	28511193	444	28510932	50	37.19	10.38	32.22	chr6.tRNA124-ArgTCG			
N74S	chr6	28556995	28557499	505	28557201	41	31.55	10.23	26.68	chr6.tRNA64-GlnTTG			
N74S	chr6	28564908	28565320	413	28565152	44	32.18	9.68	27.30	chr6.tRNA123-SerGCT			
N74S	chr6	28574848	28575174	327	28574970	33	19.68	6.99	15.04	chr6.tRNA65-AlaAGC			
N74S	chr6	28611119	28611474	356	28611266	38	24.52	7.93	19.78	chr6.tRNA66-AlaTGC			
N74S	chr6	28615781	28616256	476	28616015	65	62.83	16.65	57.43	chr6.tRNA121-ThrCGT			
N74S	chr6	28625802	28626311	510	28626056	67	65.33	17.26	59.89	chr6.tRNA120-AlaAGC			
N74S	chr6	28641458	28641857	400	28641648	65	43.94	9.57	38.85	chr6.tRNA119-AlaCGC			
N74S	chr6	28696896	28697293	398	28697090	46	32.17	9.18	27.30	chr6.tRNA70-AlaCGC			
N74S	chr6	28703131	28703409	279	28703260	21	11.80	5.67	7.31	chr6.tRNA115-ValAAC			
N74S	chr6	28710554	28710791	238	28710596	16	8.13	4.50	3.73	chr6.tRNA114-ArgCCG			
N74S	chr6	28715395	28715787	393	28715552	39	26.84	8.67	22.05	chr6.tRNA71-LysTTT			
N74S	chr6	28726078	28726402	325	28726245	34	16.37	5.50	11.80	chr6.tRNA113-AlaTGC			
N74S	chr6	28757336	28757754	419	28757572	49	45.78	14.36	40.66	chr6.tRNA110-AlaTGC			
N74S	chr6	28763528	28763959	432	28763791	49	37.76	10.89	32.78	chr6.tRNA108-AlaAGC			
N74S	chr6	28775490	28775761	272	28775652	34	17.66	5.98	13.06	chr6.tRNA106-PheGAA			
N74S	chr6	28779737	28780064	328	28779898	28	12.77	4.88	8.27	chr6.tRNA105-AlaAGC			
N74S	chr6	28784888	28785252	365	28785052	34	24.20	8.79	19.47	chr6.tRNA104-AlaTGC			
N74S	chr6	28805970	28806479	510	28806261	76	62.57	13.27	57.17	chr6.tRNA102-AlaAGC			
N74S	chr6	28831287	28831706	420	28831528	42	28.59	8.68	23.77	chr6.tRNA101-AlaAGC			

POLR1C variant	Chr	Start	End	Length	Abs summit	Pileup	-LOG10 (p-value)	FE	-LOG10 (q-value)	First Gene Full Name	Second Gene Full Name	Third Gene Full Name	Fourth Gene Full Name
N74S	chr6	28848977	28849372	396	28849219	55	51.34	15.06	46.12	chr6.tRNA73-ArgCCG			
N74S	chr6	28863832	28864300	469	28864069	90	98.00	22.88	92.34	chr6.tRNA100-LeuCAA			
N74S	chr6	28908612	28909582	971	28908834	70	65.42	16.20	59.98	chr6.tRNA74-LeuCAA	chr6.tRNA99-GlnCTG		
N74S	chr6	28911234	28911619	386	28911418	42	28.01	8.45	23.20	chr6.tRNA98-LeuAAG			
N74S	chr6	28912115	28912644	530	28912381	65	60.21	15.48	54.84	chr6.tRNA75-MetCAT			
N74S	chr6	28918642	28919035	394	28918858	76	65.72	14.41	60.28	chr6.tRNA76-LysTTT			
N74S	chr6	28920874	28921281	408	28921096	66	52.12	12.13	46.89	chr6.tRNA97-MetCAT			
N74S	chr6	28949391	28950240	850	28949998	72	55.10	11.70	49.82	chr6.tRNA96-PheGAA	chr6.tRNA77-GluCTC		
N74S	chr6	52860206	52860835	630	52860469	85	56.15	9.64	50.85	RN7SK			
N74S	chr6	126101206	126101687	482	126101455	83	63.73	12.13	58.31	chr6.tRNA87-GluCTC			
N74S	chr6	144537545	144538016	472	144537761	36	29.29	10.70	24.46	chr6.tRNA83-LeuTAA			
N74S	chr7	128423366	128423734	369	128423529	34	25.75	9.54	20.98	chr7.tRNA2-ProAGG			
N74S	chr7	139025205	139025684	480	139025438	63	38.01	8.02	33.03	chr7.tRNA3-ArgCCT			
N74S	chr7	148638411	148638861	451	148638592	69	51.70	11.25	46.48	RNY5			
N74S	chr7	148660203	148660666	464	148660388	71	50.33	10.38	45.12	RNY4			
N74S	chr7	148680680	148681050	371	148680905	44	23.61	6.47	18.88	RNY3			
N74S	chr7	148684021	148684555	535	148684330	74	45.09	8.39	39.98	RNY1			
N74S	chr8	67025418	67025847	430	67025661	53	40.56	10.95	35.53	chr8.tRNA4-TyrGTA			
N74S	chr8	67026206	67026655	450	67026375	23	15.74	7.26	11.17	chr8.tRNA5-TyrGTA	chr8.tRNA6-AlaAGC		
N74S	chr8	96281690	96282129	440	96281891	42	36.52	12.30	31.57	chr8.tRNA11-SerAGA			
N74S	chr8	124169335	124169707	373	124169559	54	46.54	13.19	41.40	chr8.tRNA10-MetCAT			
N74S	chr9	35657635	35658175	541	35657990	36	22.55	7.55	17.85	RMRP			
N74S	chr9	131102196	131102478	283	131102321	32	9.23	3.40	4.79	chr9.tRNA4-ArgTCT			
N74S	chr9	137029509	137029835	327	137029643	35	18.52	6.14	13.90	RNU6ATAC			
N74S	chr10	5895672	5895959	288	5895733	13	6.62	4.20	2.28	chr10.tRNA6-ValTAC			
N74S	chr10	22518249	22518710	462	22518491	58	48.33	12.70	43.16	chr10.tRNA4-AsnGTT			
N74S	chr10	69524056	69524546	491	69524293	60	61.73	18.14	56.34	chr10.tRNA2-SerTGA			
N74S	chr11	59317899	59319480	1582	59318103	50	40.23	11.67	35.21	chr11.tRNA17-ValTAC	chr11.tRNA16-ValTAC	chr11.tRNA3-ArgTCT	chr11.tRNA4-LeuTAA
N74S	chr11	59323660	59324124	465	59323910	42	34.99	11.55	30.07	chr11.tRNA5-LysTTT			
N74S	chr11	59324895	59325197	303	59324997	25	17.80	7.82	13.20	chr11.tRNA15-PheGAA			

POLR1C variant	Chr	Start	End	Length	Abs summit	Pileup	-LOG10 (p-value)	FE	-LOG10 (q-value)	First Gene Full Name	Second Gene Full Name	Third Gene Full Name	Fourth Gene Full Name
N74S	chr11	59327592	59328073	482	59327811	49	41.90	12.74	36.85	chr11.tRNA14-LysTTT			
N74S	chr11	59333636	59334064	429	59333861	46	42.71	13.95	37.64	chr11.tRNA13-PheGAA			
N74S	chr11	66115384	66115899	516	66115632	55	54.76	16.74	49.48	chr11.tRNA8-SerGCT			
N74S	chr11	75946339	75947160	822	75946667	88	89.83	20.14	84.22	chr11.tRNA9-ProAGG	chr11.tRNA12-ProTGG		
N74S	chr12	56583962	56584384	423	56584131	23	9.09	4.09	4.67	chr12.tRNA2-SerCGA			
N74S	chr12	96429653	96430034	382	96429861	60	38.80	8.76	33.81	chr12.tRNA4-AspGTC			
N74S	chr12	98897114	98897498	385	98897339	63	54.46	13.71	49.19	chr12.tRNA5-AspGTC			
N74S	chr12	98897815	98898283	469	98898047	64	65.07	18.09	59.63	chr12.tRNA6-TrpCCA			
N74S	chr12	125406090	125406525	436	125406333	53	43.79	12.30	38.70	chr12.tRNA13-AlaTGC			
N74S	chr12	125411697	125412651	955	125411901	82	76.34	16.60	70.81	chr12.tRNA12-AspGTC	chr12.tRNA11-PheGAA		
N74S	chr12	125423994	125424819	826	125424535	58	55.31	15.81	50.02	chr12.tRNA10-AspGTC	chr12.tRNA8-AlaTGC		
N74S	chr13	31248007	31248259	253	31248096	21	13.75	6.71	9.22	chr13.tRNA7-AsnGTT			
N74S	chr13	45491980	45492288	309	45492108	32	25.50	10.05	20.74	chr13.tRNA3-GluTTC			
N74S	chr13	95201716	95202169	454	95201941	56	53.66	15.79	48.40	chr13.tRNA1-PheGAA			
N74S	chr14	21077345	21077706	362	21077524	45	37.13	11.59	32.17	chr14.tRNA23-ProAGG			
N74S	chr14	21078094	21078618	525	21078331	66	63.88	16.98	58.46	chr14.tRNA1-LeuAAG			
N74S	chr14	21081309	21082217	909	21082014	52	34.71	8.97	29.80	chr14.tRNA22-ProAGG	chr14.tRNA21-ThrTGT		
N74S	chr14	21093341	21093948	608	21093543	60	43.20	10.22	38.12	chr14.tRNA2-LeuTAG			
N74S	chr14	21099251	21099511	261	21099371	33	14.54	4.99	10.00	chr14.tRNA20-ThrTGT			
N74S	chr14	21100980	21101433	454	21101182	42	36.52	12.30	31.57	chr14.tRNA3-ProTGG			
N74S	chr14	21149641	21150103	463	21149903	76	69.24	15.76	63.76	chr14.tRNA4-ThrTGT			
N74S	chr14	21151303	21151684	382	21151492	49	40.46	11.85	35.43	chr14.tRNA5-TyrGTA			
N74S	chr14	21152016	21152439	424	21152237	37	28.38	9.93	23.57	chr14.tRNA6-ProTGG			
N74S	chr14	23398710	23399242	533	23398933	97	111.95	26.09	106.23	chr14.tRNA7-ArgACG			
N74S	chr14	32672194	32672638	445	32672396	31	21.41	8.03	16.74	RNU6-8			
N74S	chr14	50053137	50053612	476	50053401	41	27.56	8.50	22.76	RN7SL1			
N74S	chr14	50329212	50329686	475	50329409	34	17.66	5.98	13.06	RN7SL1			
N74S	chr14	58706401	58706905	505	58706657	56	50.77	14.43	45.56	chr14.tRNA13-LysCTT			

POLR1C variant	Chr	Start	End	Length	Abs summit	Pileup	-LOG10 (p-value)	FE	-LOG10 (q-value)	First Gene Full Name	Second Gene Full Name	Third Gene Full Name	Fourth Gene Full Name
N74S	chr14	73429564	73429844	281	73429689	22	12.68	5.91	8.18	chr14.tRNA8-CysGCA			
N74S	chr14	102783216	102783679	464	102783451	65	55.32	13.45	50.04	chr14.tRNA10-IleAAT			
N74S	chr15	40885824	40886322	499	40886023	70	60.90	14.34	55.52	chr15.tRNA10-SerGCT			
N74S	chr15	45490616	45491149	534	45490817	60	61.73	18.14	56.34	chr15.tRNA9-HisGTG			
N74S	chr15	45492385	45492893	509	45492607	64	67.43	19.25	61.97	chr15.tRNA8-HisGTG			
N74S	chr15	45493158	45493607	450	45493378	39	33.84	12.00	28.94	chr15.tRNA1-HisGTG			
N74S	chr15	66161277	66161740	464	66161453	46	42.71	13.95	37.64	chr15.tRNA7-GlnCTG			
N74S	chr15	68132176	68132479	304	68132368	28	19.32	7.79	14.68	RNU6-1			
N74S	chr15	79152703	79153136	434	79152952	39	30.10	10.15	25.26	chr15.tRNA2-LysCTT			
N74S	chr15	89878055	89878611	557	89878359	101	96.36	18.36	90.71	chr15.tRNA4-ArgTCG			
N74S	chr16	686499	687013	515	686778	66	70.32	20.09	64.83	chr16.tRNA34-GlyCCC			
N74S	chr16	3200512	3200951	440	3200729	46	42.71	13.95	37.64	chr16.tRNA1-ArgCCG			
N74S	chr16	3202777	3203160	384	3202950	42	33.60	10.88	28.70	chr16.tRNA2-ArgCCT			
N74S	chr16	3207266	3207619	354	3207428	35	22.75	7.87	18.05	chr16.tRNA32-LysCTT			
N74S	chr16	3232441	3232783	343	3232683	22	12.07	5.60	7.58	chr16.tRNA29-ProAGG			
N74S	chr16	3239616	3239863	248	3239717	23	15.74	7.26	11.17	chr16.tRNA9-ProAGG			
N74S	chr16	14379591	14380086	496	14379795	51	33.69	8.81	28.79	chr16.tRNA15-ThrCGT			
N74S	chr16	22206823	22207352	530	22207062	67	71.39	20.17	65.89	chr16.tRNA27-LeuTAG			
N74S	chr16	22308221	22308823	603	22308485	69	74.71	20.92	69.19	chr16.tRNA16-LeuAAG			
N74S	chr16	57333678	57334707	1030	57334414	69	74.71	20.92	69.19	chr16.tRNA17-LeuCAG	chr16.tRNA26-LeuCAG		
N74S	chr16	70812791	70813189	399	70812972	29	19.37	7.56	14.73	chr16.tRNA24-GlyGCC			
N74S	chr16	87417488	87417850	363	87417633	33	26.66	10.33	21.87	chr16.tRNA22-MetCAT			
N74S	chr17	8022299	8023812	1514	8022536	49	46.65	14.79	41.50	chr17.tRNA2-LysTTT	chr17.tRNA3-GlnCTG	chr17.tRNA42-LeuTAG	
N74S	chr17	8028945	8029330	386	8029122	34	27.83	10.61	23.02	chr17.tRNA5-GlyGCC			
N74S	chr17	8042081	8042965	885	8042836	36	26.75	9.44	21.97	chr17.tRNA41-SerCGA	chr17.tRNA40-ThrAGT		
N74S	chr17	8089561	8091195	1635	8090969	57	57.52	17.30	52.20	chr17.tRNA6-TrpCCA	chr17.tRNA7-SerGCT	chr17.tRNA8-ThrAGT	chr17.tRNA9-IleAAT
N74S	chr17	8124022	8124393	372	8124243	35	29.01	10.89	24.18	chr17.tRNA39-TrpCCA			

POLR1C variant	Chr	Start	End	Length	Abs summit	Pileup	-LOG10 (p-value)	FE	-LOG10 (q-value)	First Gene Full Name	Second Gene Full Name	Third Gene Full Name	Fourth Gene Full Name
N74S	chr17	8124700	8126420	1721	8125602	45	41.41	13.68	36.37	chr17.tRNA10-GlyTCC	chr17.tRNA38-AspGTC	chr17.tRNA37-ProCGG	
N74S	chr17	8129435	8129667	233	8129542	20	8.72	4.35	4.30	chr17.tRNA36-ThrAGT			
N74S	chr17	19411263	19411736	474	19411486	64	53.49	13.02	48.24	chr17.tRNA12-TrpCCA			
N74S	chr17	29876901	29877416	516	29877119	50	43.13	12.98	38.06	chr17.tRNA14-ThrCGT			
N74S	chr17	36907899	36908243	345	36908066	38	29.02	9.94	24.20	chr17.tRNA31-AsnGTT			
N74S	chr17	37023743	37024206	464	37024009	32	24.46	9.51	19.73	chr17.tRNA15-CysGCA			
N74S	chr17	37025430	37025693	264	37025587	35	21.58	7.37	16.90	chr17.tRNA28-CysGCA			
N74S	chr17	37309740	37310257	518	37310018	70	69.34	17.93	63.86	chr17.tRNA27-CysGCA			
N74S	chr17	37310565	37311006	442	37310802	72	62.38	14.32	56.99	chr17.tRNA26-CysGCA			
N74S	chr17	47269669	47270151	483	47269933	73	52.51	10.65	47.27	chr17.tRNA16-GlnTTG			
N74S	chr17	66015791	66016299	509	66016046	62	40.84	9.03	35.80	chr17.tRNA23-ArgCCG			
N74S	chr17	73029867	73031447	1581	73031190	39	30.10	10.15	25.26	chr17.tRNA18-ArgCCT	chr17.tRNA21-ArgCCT	chr17.tRNA19-ArgTCG	
N74S	chr17	80452270	80452895	626	80452629	85	90.64	21.70	85.02	chr17.tRNA20-MetCAT			
N74S	chr19	893292	893732	441	893562	37	27.86	9.68	23.05	RNU6-9			
N74S	chr19	1021237	1021789	553	1021578	69	59.65	14.15	54.29	RNU6-2			
N74S	chr19	1383205	1383791	587	1383566	47	39.47	12.03	34.46	chr19.tRNA14-PheGAA	chr19.tRNA1-AsnGTT		
N74S	chr19	4723876	4724934	1059	4724095	67	71.78	20.37	66.28	chr19.tRNA2-GlyTCC	chr19.tRNA13-ValCAC		
N74S	chr19	33667751	33668208	458	33668013	45	24.49	6.61	19.76	chr19.tRNA4-ThrAGT			
N74S	chr19	45981686	45982059	374	45981928	21	13.75	6.71	9.22	chr19.tRNA8-SeCTCA			
N74S	chr21	18826934	18827353	420	18827118	61	63.14	18.41	57.73	chr21.tRNA2-GlyGCC			
N74S	chrX	18692838	18693207	370	18693037	33	26.66	10.33	21.87	chrX.tRNA4-ValTAC			
